# Antigen‐Detected NMR for Minimal Epitope Engineering and Structure‐Guided Selection of a Na_V_1.7‐Selective Nanobody

**DOI:** 10.1002/advs.77611

**Published:** 2026-09-06

**Authors:** Junyu Liu, Wanlin Chen, Ben Cristofori‐Armstrong, Theo Crawford, Kai‐En Chen, Peng Xie, Rainbow Wing Bo Chan, Yifei Zhu, Mimi Golder, Anneka Pereira Schmidt, Jennifer D. Naughton, Nicholas D. Condon, Åsa Andersson, Farhad Dehkhoda, Kirsten L. McMahon, Tabea Klasfauseweh, Ashvriya Thapa, Hue Tran, Poanna Tran, Sina Jami, Lotten Ragnarsson, Sebastian G. B. Furness, Jennifer R. Deuis, Brett M. Collins, Wai‐Hong Tham, Indira Prasadam, Irina Vetter, Mehdi Mobli

**Affiliations:** ^1^ Australian Institute for Bioengineering and Nanotechnology The University of Queensland St Lucia Queensland Australia; ^2^ Institute for Molecular Bioscience The University of Queensland St Lucia Queensland Australia; ^3^ Centre for Biomedical Technologies Faculty of Engineering Queensland University of Technology Brisbane Queensland Australia; ^4^ School of Mechanical, Medical & Process Engineering Queensland University of Technology Brisbane Queensland Australia; ^5^ The Walter and Eliza Hall Institute of Medical Research Parkville Victoria Australia; ^6^ School of Biomedical Sciences The University of Queensland St Lucia Queensland Australia; ^7^ Monash Institute of Pharmaceutical Sciences Monash University Melbourne Victoria Australia; ^8^ Department of Medical Biology The University of Melbourne Melbourne Victoria Australia; ^9^ Research School of Biology The Australian National University Canberra Australia; ^10^ School of Pharmacy and Pharmaceutical Sciences The University of Queensland Woolloongabba Queensland Australia

**Keywords:** chondrocytes, nanobody, Na_V_1.7, NMR spectroscopy, protein binder

## Abstract

Selective molecular recognition of membrane proteins is challenging because they contain few solvent‐exposed extracellular epitopes, which often depend on their native environment for structure, making them difficult to isolate faithfully for binder discovery. Here, we show that antigen‐detected NMR is well suited both to characterizing the folding of engineered minimal epitopes from the human voltage‐gated sodium channel Na_V_1.7 and to selecting binders that recognize their solvent‐exposed surfaces. Isotope labelling of the antigen enables NMR resonance assignment to assess retained local secondary structure, while ^15^N titration and zz‐exchange mapping provide binding and interface information. Combined with AlphaFold2 complex prediction, this creates a practical method for screening and ranking candidate binders. The approach was further validated by a high‐resolution x‐ray structure of an antigen–nanobody complex. Applying this workflow identified R4C8, a subtype‐ and species‐selective nanobody whose binding to the extracellular surface of human Na_V_1.7 is supported by zz‐exchange mapping, modelling, and cellular recognition, and which has minimal effects on channel gating. R4C8 detected Na_V_1.7 in engineered cell lines and in primary osteoarthritis‐derived chondrocytes, providing a useful tool for selective target detection. These results show how antigen‐detected NMR can support peptide engineering and structure‐guided protein binder selection against minimal epitopes.

## Graphical abstract

A minimal extracellular epitope from voltage‐gated sodium channel Na_V_1.7 was engineered into an isotope‐labelled antigen for antigen‐detected nuclear magnetic resonance (NMR). Together with AlphaFold2 (AF2), this enabled structure‐guided selection of R4C8, a human Na_V_1.7‐selective nanobody that labels fixed NaV1.7‐expressing cells and produces staining consistent with Na_V_1.7 recognition in osteoarthritic human chondrocytes.

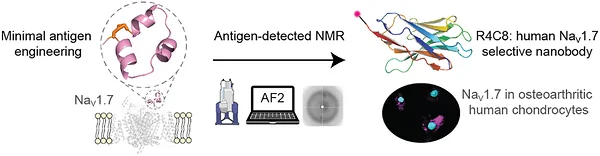

## 1 Introduction

Voltage‐gated ion channels (VGICs) are essential for electrical signaling in excitable tissues and are increasingly recognized to play important roles in non‐excitable cells, where they mediate ion flux across membranes in response to changes in membrane potential [1, 2]. This diverse family includes voltage‐gated sodium (Na_V_), potassium (K_V_), and calcium (Ca_V_) channels, each playing indispensable roles in neuronal excitability, muscle contraction, and cardiac function [3, 4, 5]. Dysregulation of VGIC activity is associated with numerous disorders, including epilepsy, cardiac arrhythmias, and chronic pain syndromes, making them prominent targets for therapeutic intervention [6, 7, 8].

Voltage‐gated sodium channel isoform 1.7 (Na_V_1.7) is a genetically and pharmacologically validated regulator of nociception, with mutations linked to congenital insensitivity to pain and a spectrum of inherited pain disorders [4, 9, 10]. In addition, Na_V_1.7 has been implicated in anosmia, autonomic dysfunction, cancer, and more recently, in the pathophysiology of COVID‐19‐related pain and osteoarthritis [2, 11, 12, 13, 14]. Ligands that selectively target Na_V_1.7 are therefore valuable not only as molecular probes to dissect channel structure and function but also as potential therapeutic leads [15, 16, 17].

Nanobodies, the antigen‐binding fragments of camelid heavy‐chain‐only antibodies, represent an emerging class of ligands distinguished by their high affinity, specificity, and adaptability. They also offer several advantages over conventional antibodies, including improved access to restricted epitopes, better tissue penetration, and relative ease of production [18, 19]. These features have made nanobodies powerful tools for studying G protein‐coupled receptors (GPCRs) and enzymes, where they have been used to stabilize transient conformations for structural studies and to support the development of novel therapeutic modalities [20, 21].

A widely used route to nanobody discovery is immunization of alpacas, which depends critically on the availability of high‐quality antigens that faithfully reproduce the native conformation of the target [18, 22]. This presents a major challenge for Na_V_1.7, whose complex topology offers only a limited number of solvent‐exposed extracellular regions. Although successful immunization has been reported using recombinant cells that display the membrane protein of interest, this approach requires substantial downstream screening to eliminate nanobodies that bind unintended targets on the cell surface [23, 24]. A more direct strategy for developing Na_V_1.7‐selective nanobodies is therefore to target solvent‐exposed extracellular regions that differ from other Na_V_ isoforms. These regions are largely confined to a few short extracellular loops with limited secondary structure, which may not fold correctly when removed from the channel. To improve the immunogenicity and structural integrity of short peptide antigens, such sequences have previously been grafted onto scaffold proteins or presented in complex with partner proteins [25]. More recently, strategies employing scaffolded proteins in display systems have also shown promise when screening in vitro libraries without immunization [26]. However, these approaches still permit enrichment of binders to unrelated scaffold surfaces, which reduces the efficiency of identifying nanobodies directed against the intended extracellular epitope.

Here, we show how NMR can be used both to guide the engineering of minimal antigens that retain native‐like structural features and to support the downstream selection of suitable binding proteins. We term this strategy “antigen‐detected NMR” and apply it to the development of a highly selective nanobody against the human Na_V_1.7 channel (Figure S1). Our results show that introduction of a disulfide bond that links the ends of one Na_V_1.7 pore loop helps preserve native‐like secondary structure, improves serum stability, and is associated with successful immunization in alpacas. Isotope labelling of the engineered antigen enables multidimensional heteronuclear NMR experiments for resonance assignment, while ^15^N titration and zz‐exchange measurements provide residue‐level information on binding interfaces and relative interaction strength. When combined with AlphaFold2 predictions, this creates a practical medium‐throughput method for prioritizing candidate nanobodies for detailed characterization by ITC and x‐ray crystallography. Using this workflow, we identified R4C8 from a nanobody library as a subtype‐ and species‐selective nanobody that recognizes human Na_V_1.7 with minimal effects on channel function. R4C8 further provides a probe for selective detection of Na_V_1.7 in transfected cell lines and primary human chondrocytes. Together, these findings present antigen‐detected NMR as a useful strategy for selecting protein binders against solvent‐exposed loops from challenging membrane proteins.

## 2 Results and Discussion

### 2.1 Minimal Epitope Selection and Engineering Yield a Stabilized Na_V_1.7 Antigen

**FIGURE 1:**
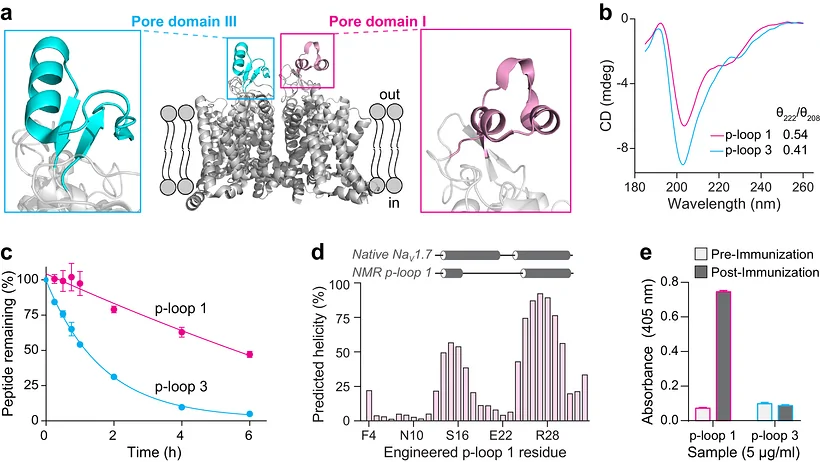
Stabilized extracellular loops of Na_V_1.7 enable nanobody generation. (a) Side view of human Na_V_1.7 (PDB ID: 6J8G) with extracellular regions of pore domain I (right) and III (left) expanded. (b) Overlay of circular dichroism (CD) spectra of p‐loop 1 (magenta) and p‐loop 3 (cyan). All spectra were recorded at a concentration of 20 µM in 10 mM phosphate buffer, pH 7.4. (c) Serum stability assay for p‐loop 1 (magenta) and p‐loop 3 (cyan). After incubating with mouse serum, the remaining p‐loop was quantified by LC‐MS and normalized to the zero time point. Data points represent the average ± SEM of triplicate experiments. (d) NMR chemical shift‐based (TALOS‐N) analysis of helical propensity across p‐loop 1. Helical regions in the native and engineered p‐loop 1 are annotated based on experimentally validated structure (PDB ID: 6J8G, ‘native’) and chemical shifts (‘NMR’), respectively. The *x*‐axis is labelled according to the sequence of the labelled p‐loop 1 (Figure S2e). (e) Reactivity of serum from p‐loop 1‐immunised alpaca against p‐loop 1 (left) and p‐loop 3 (right). Microtiter wells were coated with biotinylated p‐loop as indicated (*x*‐axis). Binding of alpaca serum was detected using Horseradish peroxidase (HRP)‐conjugated anti‐alpaca secondary antibodies, with absorbance measured at 405 nm (technical duplicates).

Na_V_1.7 (UniProt ID: Q15858; Figure 1 and Figure S2) contains two relatively large and continuous extracellular regions in pore domain I (native p‐loop 1) and pore domain III (p‐loop 3). These regions were selected based on their defined secondary structures and their unique amino acid sequences relative to other Na_V_ isoforms (Figure S2a,b), making them attractive candidate epitopes for generating subtype‐selective nanobodies.

For nanobodies raised against isolated pore‐domain peptides to recognize the full‐length channel, these peptides must retain native‐like conformations in solution. AlphaFold2 predictions indicated that isolated p‐loop 3 largely maintains a native‐like conformation (Figure S2f), whereas native p‐loop 1 adopted a different conformation when modelled in isolation (Figure S2h). Because p‐loop 3 contains a native intramolecular disulfide bond while p‐loop 1 does not, we reasoned that introducing a disulfide constraint might stabilize p‐loop 1 in a native‐like state (Figure S2e,g). We therefore engineered a cysteine at the C‐terminus of p‐loop 1 (corresponding to a Y304C mutation), designed to form an intramolecular disulfide bond with the cysteine corresponding to position C275 (Figure S2e). AlphaFold2 prediction of this engineered construct suggested that the disulfide‐stabilized peptide adopts a conformation more closely resembling that of p‐loop 1 in the full‐length channel (Figure S2g). This construct is referred to hereafter as p‐loop 1.

### 2.2 Structural and Immunological Qualification Identifies p‐Loop 1 as a Suitable Minimal Antigen

P‐loop 1 and p‐loop 3 were produced recombinantly, and their secondary structures were first assessed by circular dichroism (CD) spectroscopy (Figure 1b and Figure S3a,b). P‐loop 1 displayed a strong negative signal near 190 nm, a pronounced minimum near 205 nm, and a weaker minimum around 222 nm, consistent with a mixture of helical structure and random coil. In contrast, p‐loop 3 showed a deeper minimum around 200 nm without a discernible minimum at 222 nm, indicative of a predominantly disordered conformation with little helical content. Consistent with this, p‐loop 1 displayed a larger θ_222_/θ_208_ ratio, supporting a greater helical contribution. These data suggest that the engineered disulfide in p‐loop 1 acts primarily as a conformational staple that restricts the relative arrangement of structural elements, rather than by directly increasing intrinsic helicity.

Because sustained antigen integrity is likely to favor productive immune recognition [27], we next compared the serum stability of both p‐loops by incubating them in mouse serum and analyzing the remaining peptide by LC‐MS. After 6 h, approximately 50% of p‐loop 1 remained, compared with less than 10% of p‐loop 3, indicating substantially greater stability of p‐loop 1 (Figure 1c). Thus, relative to p‐loop 3, p‐loop 1 combines greater helical character with improved serum stability.

To determine whether p‐loop 1 retained native‐like local secondary structure in solution and to establish whether it would be suitable for antigen‐detected NMR, we next produced uniformly ^13^C/^15^N‐labelled p‐loop 1 and assigned its backbone resonances using multidimensional heteronuclear NMR spectroscopy with fast acquisition and non‐Fourier reconstruction methods (Figure S3c) [28, 29, 30]. Backbone dihedral angles predicted from secondary chemical shifts using TALOS‐N indicated that residues L14–S16 and E24–Y30 are consistent with α‐helical structure (Figure 1d) [31]. These findings agree with the CD data and AlphaFold2 predictions, and show that the disulfide‐stabilized construct retains native‐like secondary‐structure features in solution. Importantly, the small size and defined fold of p‐loop 1 also made it amenable to recombinant isotope labelling and backbone assignment, establishing it as suitable for antigen‐detected NMR.

We next evaluated whether these engineered epitopes were immunologically competent in vivo. Two adult alpacas were immunized separately with purified p‐loop 1 and p‐loop 3 according to established protocols [32]. Only p‐loop 1 elicited a detectable and selective immune response. Serum from;loop 1‐immunized animal reacted with p‐loop 1 but not with p‐loop 3, indicating that the immune response was epitope‐specific (Figure 1e). Because p‐loop 3 did not yield a detectable selective immune response, we did not pursue it further.

Together, these data identify disulfide‐stabilized p‐loop 1 as the more suitable minimal antigen. In contrast to p‐loop 3, p‐loop 1 retained native‐like secondary‐structure features, showed improved serum stability, and elicited a selective immune response in alpacas. While these comparisons do not establish general rules for minimal antigen design, they show that preserved secondary structure and improved serum stability were associated with successful campaign outcome in this system [33, 34, 35, 36, 37, 38, 39, 40, 41, 42, 43].

### 2.3 Nanobody Discovery Against p‐Loop 1 Yields 14 Sequence‐Diverse Candidates

We therefore advanced p‐loop 1 into nanobody discovery and constructed both an immunization‐derived phage‐display library and a larger complementary proprietary library assembled from previous camelid immunization campaigns, containing 10^5^ unique nanobody clones, to broaden the diversity of candidate binders. Both libraries were selected against p‐loop 1, immobilized either by direct coating or by biotin–neutravidin capture, with counter‐selection against the homologous Na_V_1.6 p‐loop 1 to enrich for target‐specific binders (Figure S4a,b). Across these campaigns, 188 clones were screened by ELISA, yielding 122 positive hits (Figure S4c). Sequencing identified 14 nanobody sequences with diverse complementarity‐determining region 3 (CDR3) sequences (Figure S4d and Table S1).

### 2.4 Antigen‐Detected NMR and AlphaFold2 Triage Identify Four Lead Nanobodies

In the isolated peptide, p‐loop 1 presents both the extracellularly accessible face of the native epitope and a face that is buried in the full‐length channel. Affinity alone is therefore insufficient to identify nanobodies likely to recognize Na_V_1.7 in its native membrane context. To address this, we used NMR as an initial triage step and integrated the resulting data with AlphaFold2 multimer modelling. 12 of 14 nanobodies were produced recombinantly for antigen‐detected NMR screening. R4A5 and R3E6 were excluded due to their predicted isoelectric points being close to physiological pH, which could complicate downstream applications.

**FIGURE 2:**
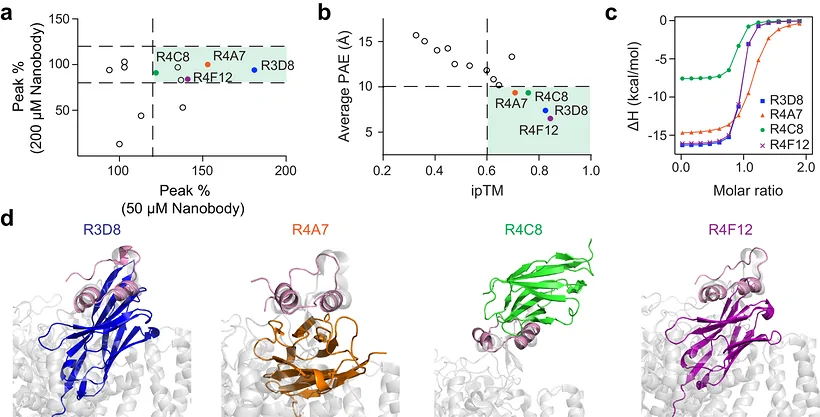
Integrative refinement of nanobody candidates to target the extracellular surface of Na_V_1.7. (a) Quantitation of slow exchange observed in ^1^H‐^15^N HSQC spectra. The change in the number of observable peaks from ^15^N‐labelled p‐loop 1 (100 µm) upon titration with each of the 12 nanobodies was used to calculate a ratio of observed peaks in the absence and presence of each nanobody, under sub‐saturating (50 µm, *x*‐axis) and saturating (200 µm, *y*‐axis) nanobody concentrations. The green zone indicates high‐affinity binding, and those with high‐confidence AlphaFold2 predictions are labelled (see panel b). (b) AlphaFold2 predictions of nanobody:p‐loop 1 complexes, plotted by interface predicted TM‐score (ipTM) and average inter‐chain predicted aligned error (PAE). Nanobodies within the green zone (ipTM > 0.6 and PAE < 10 Å) were considered reliable and labelled. (c) Isothermal titration calorimetry (ITC) data for nanobodies R3D8 (blue, *K*_D_ = 64.4 ± 7.6 nm), R4A7 (orange, *K*_D_ = 256.0 ± 14.1 nm), R4C8 (green, *K*_D_ = 86.5 ± 8.0 nm), and R4F12 (purple, *K*_D_ = 67.3 ± 3.2 nm) titrated into p‐loop 1. (d) Structural alignment of AlphaFold2‐predicted nanobody:p‐loop 1 complexes (nanobodies colored as in c; p‐loop 1 in pink) with the full‐length Na_V_1.7 channel (PDB: 6J8G, in grey). R3D8, R4A7, and R4F12 are predicted to bind surfaces that are buried in the native channel, whereas R4C8 is predicted to bind an exposed extracellular region.

We first used antigen‐detected ^15^N‐HSQC titrations to identify strongly binding nanobodies. Each candidate nanobody was titrated into ^15^N‐labelled p‐loop 1, and the resulting spectral changes were monitored. At a sub‐saturating p‐loop 1:nanobody ratio of 1:0.5 (100 µm p‐loop 1:50 µm nanobody), seven nanobodies (R3D8, R4A7, R4C8, R4C9, R4E9, R4F12, and R4G10) showed clear slow‐exchange behavior, consistent with strong binding. At a saturating nanobody concentration of 200 µm (1:2 ratio), a distinct bound‐state spectrum was observed for all except R4E9 (Figure 2a and Figures S5–S7 and Table S2). In the case of R4E9, substantial peak broadening was observed under saturating conditions, suggesting aggregation, and this nanobody was therefore excluded from further analysis. Taken together, antigen‐detected NMR titration reduced the panel to six high‐affinity candidates: R3D8, R4A7, R4C8, R4C9, R4F12, and R4G10.

In parallel, we used AlphaFold2 multimer to predict nanobody:p‐loop 1 complexes and assessed each model using the interface‐predicted TM‐score (ipTM) and the average inter‐chain predicted aligned error (PAE) [44, 45]. The ipTM score ranges from 0 to 1, with higher values indicating greater confidence in the predicted protein–protein interface. Here, we used an ipTM threshold of 0.6, based on guidance from the AlphaFold developers that predictions with ipTM < 0.6 are more likely to represent failed or unreliable interaction models; this threshold has consequently been adopted as a practical guideline in other recent studies [46]. In parallel, PAE provides an estimate of the confidence in the relative positioning of residues or chains within the predicted complex, with lower PAE values indicating higher confidence. In this study, we used an average inter‐chain PAE threshold of 10 Å as an empirical cutoff to prioritize models with more confidently defined interfaces. Filtering the prediction with the two criteria yielded nanobodies—R3D8, R4A7, R4C8, and R4F12—that met these criteria (Figure 2b), and all four were also highlighted by the antigen‐detected NMR titrations. Isothermal titration calorimetry (ITC) confirmed that these four nanobodies bind p‐loop 1 with nanomolar dissociation constants (*K*_D_; Figure 2c and Figure S8).

To determine whether these nanobodies were likely to recognize the native extracellular surface of Na_V_1.7, we aligned the AlphaFold2‐predicted nanobody:p‐loop 1 complexes with the full‐length Na_V_1.7 structure (PDB ID: 6J8G). This analysis suggested that R3D8, R4A7, and R4F12 engage faces of p‐loop 1 that are buried or sterically inaccessible in the full‐length channel, whereas R4C8 targets the extracellularly accessible interface (Figure 2d). Thus, although several nanobodies bound tightly to the isolated epitope, only R4C8 was predicted to recognize the face of p‐loop 1 that remains exposed in native Na_V_1.7.

### 2.5 Interface Mapping Prioritizes R4C8 for Recognition of Native Na_V_1.7

**FIGURE 3:**
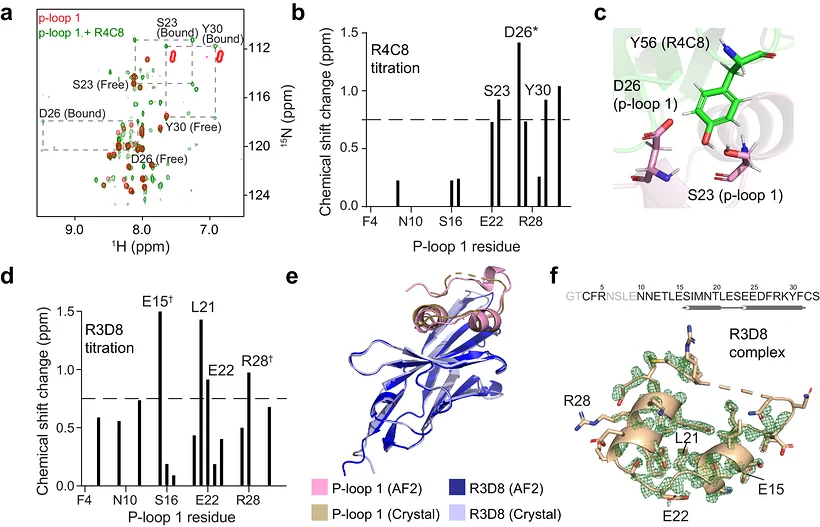
Structural details of nanobody – Na_V_1.7 p‐loop 1 complexes. (a) Overlay of HSQC (p‐loop 1, red) and R4C8 zz‐exchange spectra (green, p‐loop 1 + 50 µm R4C8). Dashed lines mark bound and unbound states of key residues. (b) Chemical shift perturbations across p‐loop 1 in R4C8 zz‐exchange experiment. Residues above the threshold (dashed line) are labelled. D26 is solvent exposed in its native state. The last residue above the threshold is the engineered C32. (c) AlphaFold2‐predicted interactions between p‐loop 1 (pink) key residues and R4C8 (green). (d) Chemical shift perturbations across p‐loop 1 residues in R3D8 zz‐exchange experiment. E15 and R28 are inaccessible in the full‐length channel (marked with daggers). (e) Structural alignment of the crystal (PDBID: 9Y7F) and AlphaFold2‐predicted R3D8:p‐loop 1 complex (backbone RMSD = 0.3 Å). (f) Electron density of p‐loop 1 corresponding to a simulated‐annealing OMIT Fo‐Fc map contoured at 3σ.

We next used antigen‐detected zz‐exchange experiments to validate the predicted nanobody:p‐loop 1 complexes directly in solution. Under fast exchange, ^15^N titration may be sufficient to map binding interfaces [47], however, for high‐affinity binding to minimal epitopes zz‐exchange becomes necessary for transferring assignments to the bound state. By comparing the free and bound resonances of ^15^N‐labelled p‐loop 1, this approach provided residue‐level epitope information that could be compared directly with the AlphaFold2 models. For R4C8, zz‐exchange mapping showed strong agreement with the predicted complex (Figure 3a,b and Figure S9). The largest chemical shift changes were observed for residues centered on E22, D26, F27, and Y30 of p‐loop 1, supporting a binding mode in which R4C8 engages the extracellularly exposed face of the epitope. In the AlphaFold2 model, Y56 in CDR2 of R4C8 is positioned to participate in a hydrogen‐bonding network involving S23 and D26 of p‐loop 1 (Figure 3c). Additional contacts include interactions between K32 in CDR1 of R4C8 and E22, D26, and F27 in p‐loop 1, while Y30 at the C‐terminal end of the helical region is positioned in a hydrophobic pocket formed by V2, L31, and I104 of R4C8 (Figure S9). Notably, D26, which showed the largest perturbation and appears to make a major contribution to binding, is solvent‐exposed in the full‐length channel. More broadly, the key perturbed residues cluster within and adjacent to the helical region of p‐loop 1, consistent with the idea that preserving this structured face was important both for antigen design and for productive binder selection.

Of the three nanobodies predicted to bind the channel‐occluded face of p‐loop 1, R3D8 showed the strongest affinity by ITC and the most favorable AlphaFold2 confidence metrics. Antigen‐detected zz‐exchange mapping of the R3D8:p‐loop 1 complex revealed substantial perturbations at residues E15, L21, E22, and R28 (Figure S9), again in good agreement with the predicted interface. Importantly, we were able to determine the crystal structure of the R3D8:p‐loop 1 complex (Figure 3e,f and Table S3). The experimental structure closely matched the AlphaFold2 model, with a backbone RMSD of 0.3 Å, and confirmed that R3D8 binds a face of p‐loop 1 that is occluded in full‐length Na_V_1.7. In the crystal structure, N54 and S57 in CDR2 of R3D8 form hydrogen bonds with the side chain of E15 in p‐loop 1, R59 engages the backbone of L21 and E22, and E44 forms a potential salt bridge with R28. Zz‐exchange mapping of the two additional nanobodies predicted to bind the buried face also supported binding to this channel‐inaccessible interface (Figure S10), indicating that the solution‐state interfaces were broadly consistent with the computationally predicted complexes (see also summary in Table S4).

Although crystallographic validation could only be obtained for the R3D8:p‐loop 1 complex, the strong agreement between AlphaFold2 prediction, antigen‐detected zz‐exchange mapping, and x‐ray crystallography supports the broader logic of the workflow. In this framework, antigen‐detected ^15^N‐HSQC titrations act as an initial experimental filter to identify binders that genuinely form complexes with the minimal epitope in solution, whereas zz‐exchange mapping provides residue‐level information that can be used to discriminate between competing structural models of those complexes. This distinction is important because current computational methods are highly effective at generating candidate binder:target models, but substantially less reliable in establishing which predicted complexes are realized experimentally [48, 49]. Recent studies evaluating the performance of AlphaFold in modelling nanobody–antigen interactions have also demonstrated that the method can generate structurally plausible complex models, particularly for solvent‐accessible epitopes, while also highlighting limitations in accurately resolving binding interfaces and in interpreting confidence metrics as indicators of binding strength or specificity [46]. Notably, when we extended AlphaFold‐based modelling to the full‐length Na_V_1.7 channel, both AlphaFold2 and AlphaFold3 failed to converge on a consistent nanobody binding mode. This is in line with known limitations of current prediction methods for large, multi‐domain membrane proteins, and further highlights the importance of combining structure prediction with experimental validation, and a potential benefit in minimal epitope modelling [50]. Antigen‐detected NMR therefore addresses two linked problems in binder discovery: it identifies which candidates bind in solution, and it helps determine how they bind. In cases where multiple models are generated by AlphaFold2, RF Diffusion design pipelines, or alternative ranking criteria [51], zz‐exchange data provides an efficient experimental filter for selecting models that are consistent with the observed solution complex. Based on the results of the antigen‐detected NMR workflow, we therefore prioritized R4C8 as the candidate most likely to recognize the native extracellular surface of full‐length Na_V_1.7.

### 2.6 R4C8 Selectively Recognizes Human Na_V_1.7 in Fixed Cells

**FIGURE 4:**
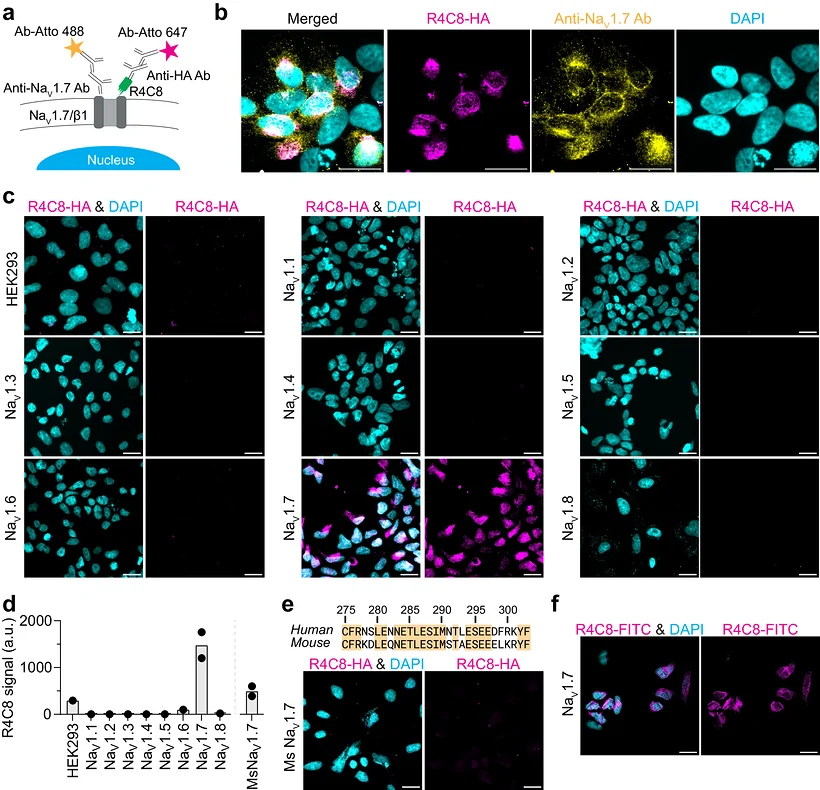
R4C8 selectively labels cells expressing human Na_V_1.7 with subtype and species specificity. (a) Schematic of dual‐labelling strategy in HEK293‐Na_V_1.7/β1 cells using R4C8‐HA and a commercial anti‐Na_V_1.7 antibody. (b) Representative confocal images showing co‐labeling of HEK293‐Na_V_1.7/β1 cells with R4C8‐HA (detected with anti‐HA/Atto 647) and a commercial anti‐Na_V_1.7 antibody (detected with Atto 488). Panels (left to right): merged image, Atto 647 channel, Atto 488 channel, and DAPI channel. (c) Representative confocal images of R4C8‐HA staining in control HEK293 cells and HEK293 cells expressing individual human Na_V_ isoforms (Na_V_1.1–Na_V_1.7; note Na_V_1.8 in CHO cells). Each panel shows: merged image (left) and Atto 647 channel (right). (d) Quantification of R4C8‐HA staining in parental HEK293 cells and HEK293 cells expressing the indicated human Na_V_ isoforms or mouse Na_V_1.7. For each field, Cy5‐channel integrated fluorescence intensity was corrected using the mean intensity of three cell‐free background regions and normalized to the number of DAPI‐positive nuclei. Points represent individual imaged fields and bars indicate the mean. Values are reported as background‐corrected R4C8 signal per cell in arbitrary units; underlying measurements and cell numbers are provided in Table S5. (e) Left: Representative confocal images of R4C8‐HA staining in HEK293 cells expressing Ms Na_V_1.7. Right: Sequence alignment of human and mouse Na_V_1.7 p‐loop 1. Identical residues are highlighted in dark yellow (human Na_V_1.7 numbering, UniProt ID: Q15858). (f) Representative confocal images of HEK293‐Na_V_1.7/β1 cells stained with R4C8‐FITC. All scale bars: 20 µm.

Structural alignment predicted that R3D8, R4A7, and R4F12 engage the channel‐facing surface of p‐loop 1, whereas R4C8 engages the extracellularly accessible surface. R4C8 was therefore prioritised for cellular validation. Cellular recognition by the other candidates was not resolved in this study. To assess the binding of R4C8 to Na_V_1.7 in a native membrane context, we performed immunofluorescent staining in HEK293 cells stably expressing human Na_V_1.7 and its auxiliary β1 subunit (HEK293‐Na_V_1.7/β1). Fixed cells were stained using R4C8 with a C‐terminal influenza hemagglutinin tag (R4C8‐HA), followed by detection with an anti‐HA antibody and a fluorescently labelled secondary antibody (Atto 647‐conjugated; Figure 4a). In parallel, cells were stained with a commercially available anti‐Na_V_1.7 antibody (ASC‐027, Alomone Labs) that recognizes a distinct extracellular epitope, detected using an Atto 488‐conjugated secondary antibody. Co‐localization of the two signals was observed in HEK293‐Na_V_1.7/β1 cells, indicating that R4C8‐HA and the commercial antibody recognize the same target protein (Figure 4b). Pixel‐wise quantitative analysis yielded a Pearson's correlation coefficient of 0.74. The Costes thresholded Manders’ coefficient tM1 was 0.634, indicating that 63.4% of the R4C8 signal overlapped with the anti‐Na_V_1.7 antibody signal, while tM2 was 0.379, corresponding to 37.9% of the antibody signal overlapping with R4C8. Taken together, these results confirm co‐localization.

We next assessed the subtype selectivity of R4C8 across a panel of cell lines expressing individual human Na_V_ isoforms (Na_V_1.1–1.6, co‐expressed with β1; Na_V_1.8, co‐expressed with β3, Figure 4). Robust R4C8 staining was observed exclusively in cells expressing human Na_V_1.7, with no detectable signal for other isoforms (Figure 4c,d). As all Na_V_1.1–1.6 lines co‐express β1, these results indicate that binding is driven by the Na_V_1.7 α‐subunit. Functional expression of the intended NaV isoforms in these cell lines was confirmed by whole‐cell current recordings (Figure S11).

To evaluate species specificity, we also stained HEK293 cells stably expressing mouse Na_V_1.7 (Ms Na_V_1.7). Despite the considerable sequence conservation between human and mouse Na_V_1.7 p‐loop 1 (20/29 identity), R4C8 staining in mouse Na_V_1.7‐expressing cells was markedly lower than that observed for human Na_V_1.7, consistent with species‐selective recognition. (Figure 4d,e).

We next asked whether a fluorescent probe could be conjugated directly to R4C8 to enable single‐step detection. Fluorescein isothiocyanate (FITC) was site‐specifically conjugated to the C‐terminus of R4C8 via Sortase A ligation (R4C8‐FITC, Figure S12) [52]. Bright fluorescence was observed in HEK293‐Na_V_1.7/β1 cells stained with R4C8‐FITC (Figure 4f). These data show that R4C8‐FITC can label cells overexpressing Na_V_1.7 in a single staining step, making it a practical probe for imaging applications. Finally, we note that all cellular imaging in this study was performed on fixed, non‐permeabilized cells. Although the mapped R4C8 interface is not centred on residues expected to be most reactive during formaldehyde fixation, fixation‐dependent changes in epitope accessibility cannot be excluded. Validation and quantitative analysis on unfixed cells will require a more sensitive high‐expression assay platform.

Although R4C8 binds the isolated p‐loop 1 antigen with nanomolar affinity and selectively labels fixed, non‐permeabilized Na_V_1.7‐expressing cells, we were unable to derive a reliable apparent affinity for full‐length Na_V_1.7 under the live‐cell flow‐cytometry conditions tested. At low nanobody concentrations, the specific signal was below the assay detection window, whereas higher concentrations produced increasing background in parental cells. The affinity of R4C8 for full‐length Na_V_1.7 therefore remains to be established using a higher‐expression or more sensitive cellular platform (Figure S13).

Together, these results show that R4C8 selectively labels human Na_V_1.7 in fixed, non‐permeabilized cells and can be adapted for fluorescence‐based detection.

### 2.7 R4C8 Has Minimal Effects on Na_V_1.7 Gating

**FIGURE 5:**
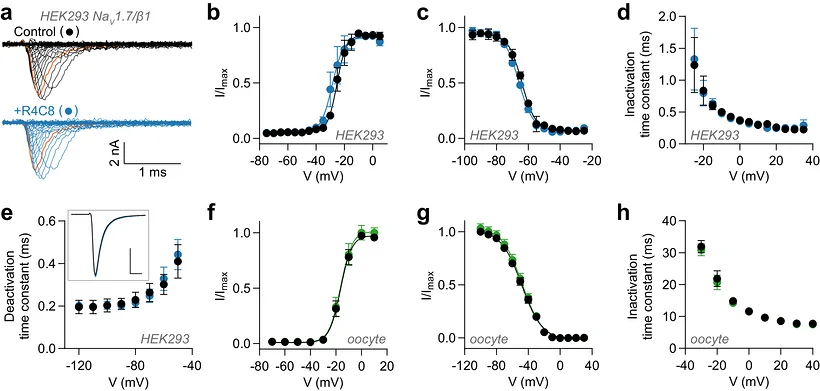
Electrophysiological assessment of R4C8 at human Na_V_1.7 in HEK293 cells and *Xenopus* oocytes. (a) Representative whole‐cell patch‐clamp traces from HEK293‐Na_V_1.7/β1 cells showing sodium currents before (Control; black) and after application of 10 µm R4C8 (blue). Currents elicited by a voltage pulse to −10 mV are highlighted in orange. (b) Conductance‐voltage (G–V) relationships in control (black; V_50_ = −25.3 mV, 95% CI: −26.4–−24.2) and with 10 µm R4C8 (blue; V_50_ = −29.7 mV, 95% CI: −30.5–−26.8) (*n* = 4). (c) Steady‐state fast inactivation curves from HEK293‐Na_V_1.7/β1 cells in control (black; V_50_ = −64.1 mV, 95% CI: −65.1–−63.0) and after application of 10 µm R4C8 (blue; V_50_ = −66.0 mV, 95% CI: −66.8–−65.2) (*n* = 4). (d) Inactivation time constants measured from HEK293‐Na_V_1.7/β1 I‐V data in control (black) and R4C8 (blue) (*n* = 4). No significant differences were observed at any voltage (P = 0.959, two‐way ANOVA). (e) Representative deactivation tail currents in control (black) and after the addition of 10 µM R4C8 (blue) from manual patch‐clamp recordings of HEK293‐Na_V_1.7/β1 cells (*n* = 12). No significant differences were observed at any voltage (P = 0.999, two‐way ANOVA). Grey box inset shows representative deactivation tail currents in control (black) and with R4C8 (blue) from a depolarization pulse to −10 mV for 0.5 ms, then −50 mV (Vh = −120 mV). Scale bar: abscissa 1 ms, ordinate 1 nA. (f) Conductance‐voltage (G‐V) relationship from Na_V_1.7 expressed in oocytes in control (black; V_50_ = −16.4 mV, 95% CI: ‐17.8 to ‐14.9) and with 10 µm R4C8 (green; V_50_ = −16.4 mV, 95% CI: −18.5–−14.2) (*n* = 8). (g) Steady‐state fast inactivation curves from Na_V_1.7 in oocytes in control (black; V_50_ = −48.3 mV, 95% CI: −49.9–−46.7) and with 10 µm R4C8 (green; V_50_ = −47.7 mV, 95% CI: −50.5–−45.1) (*n* = 8). (h) Inactivation time constants measured from oocyte I‐V data in control (black) and with 10 µm R4C8 (blue) (*n* = 8). No significant differences were observed at any voltage (P = 0.459, two‐way ANOVA). Data points represent the average ± SEM.

We next investigated whether R4C8 binding alters the biophysical properties of Na_V_1.7, given that p‐loop 1 is located near the ion‐conducting pore and may influence channel gating, and because a nanobody independently reported to target the same region was shown to modulate Na_V_1.7 deactivation kinetics [26]. Using automated whole‐cell patch‐clamp electrophysiology in HEK293‐Na_V_1.7/β1 cells, we assessed voltage‐dependent gating properties of Na_V_1.7 in the absence (control) or presence of 10 µm R4C8. Application of R4C8 did not affect peak current amplitude elicited by a voltage pulse to −10 mV (I_R4C8_/I_Control_ = 0.93, 95% CI: 0.84−1.03; Figure 5a,b) and did not significantly shift the voltage dependence of activation (Figure 5b and Figure S14a,b). R4C8 produced a small, statistically significant shift in steady‐state fast inactivation (ΔV_50_ = −1.9 mV; P  = 0.021), though this difference is unlikely to be biologically meaningful (Figure 5c and Figure S14c). No significant differences were observed in the time constants of inactivation between −25 and 35 mV (Figure 5d). Deactivation kinetics measured by manual patch‐clamp also showed no significant differences between control and R4C8‐treated cells (Figure 5e).

To assess R4C8 activity using an independent electrophysiological platform, we performed two‐electrode voltage‐clamp recordings in *Xenopus laevis* oocytes expressing only the Na_V_1.7 α‐subunit. Consistent with patch‐clamp data, R4C8 had no measurable effect on peak current amplitude, voltage‐dependent activation, steady‐state fast inactivation, or the time constants of inactivation in oocytes (Figure 5f–h and Figure S14d–g).

While this work was being completed, Martina et al. independently reported a nanobody targeting the same p‐loop 1 region of Na_V_1.7 [26]. Although both studies validate this region as a viable extracellular epitope, the antigen design and discovery strategies differ substantially. Martina et al. employed a larger, scaffolded antigen for in vitro library screening and identified a nanobody that modulates channel deactivation kinetics with cross‐species reactivity. In contrast, our approach used a minimal, structurally stabilized peptide antigen and yielded R4C8, a human‐selective nanobody with no measurable effects on Na_V_1.7 gating in the electrophysiological assays used here. These differences are consistent with distinct binding interfaces and mechanistic outcomes. R4C8 therefore provides a useful molecular probe for studying Na_V_1.7 distribution and extracellular recognition with minimal effects on channel function, which may be valuable in the context of recent reports linking Na_V_1.7 inhibition to cardiac effects [12].

### 2.8 R4C8 Detects Na_V_1.7 in Primary Human Chondrocytes

**FIGURE 6:**
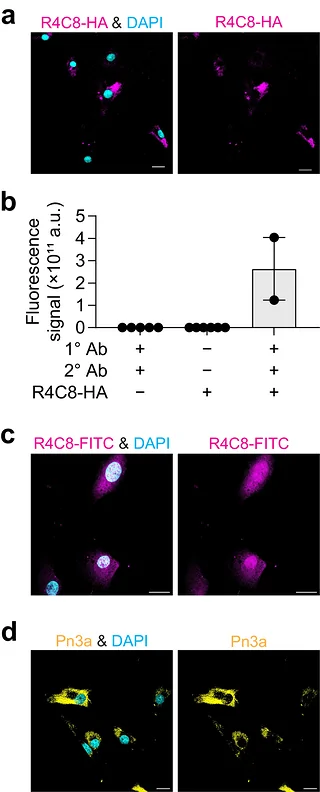
Detection of Na_V_1.7 in human osteoarthritis chondrocytes using R4C8 and Pn3a. (a) Representative R4C8‐HA staining with merged image (left) and Atto 647 channel (right). Scale bar: 50 µm. (b) Quantification of fluorescence staining in primary human chondrocytes treated with R4C8‐HA, compared with detection‐antibody‐only and R4C8‐HA‐without‐detection‐antibody controls. Integrated fluorescence intensity was corrected using the mean intensity of three signal‐free background regions and normalized to the number of DAPI‐positive nuclei. Points represent individual coverslips, and bars indicate the mean. (c) As in panel a, showing R4C8‐FITC staining. Scale bar: 20 µm. (d) As in panel a, showing biotin‐Pn3a staining using streptavidin Atto‐488. Scale bar: 20 µm.

Na_V_1.7 has recently been implicated in osteoarthritis, with its expression inferred from transcriptomic analysis and electrophysiology [2]. To determine whether R4C8 can detect Na_V_1.7 in primary human cells, we performed immunofluorescence staining on fixed chondrocytes derived from osteoarthritis patients using R4C8‐HA and R4C8‐FITC. Staining with the same protocols used for HEK293‐Na_V_1.7/β1 cells revealed membrane‐associated fluorescence, consistent with surface expression of Na_V_1.7 (Figure 6a–c and Figure S15). To further support the specificity of R4C8 staining, we compared the labelling with 100 nm biotin‐conjugated Pn3a, a well‐characterized Na_V_1.7‐binding peptide, and detected via streptavidin–Alexa Fluor 488. Both R4C8‐HA and Pn3a produced similar staining patterns in OA chondrocytes, consistent with R4C8 recognition of NaV1.7 in these cells.

While R4C8 produced membrane‐associated staining in fixed osteoarthritis‐derived chondrocytes, with a distribution similar to that observed using the independently validated Na_V_1.7 ligand Pn3a, the low target abundance and lack of a robust live‐cell competition window limit quantitative interpretation. Further optimization will be required for sensitive quantification in primary cells.

Together, these results demonstrate that R4C8 staining of fixed primary human chondrocytes is consistent with the expected Na_V_1.7 expression in these cells, highlighting its potential as a molecular tool for studying Na_V_1.7 distribution in disease‐relevant tissues or as a targeting module for ligand delivery [53].

All procedures involving the immunization and handling of alpacas were approved by the Agriculture Victoria Wildlife & Small Institutions Animal Ethics Committee (project approval No. 36.20). All procedures involving human chondrocytes were approved by the ethics committees of Queensland University of Technology and St Vincent Private Hospital, and all participants provided informed consent (ethics number: #1400001024). Experiments involving Xenopus laevis oocytes were approved by The University of Queensland Animal Ethics Committee (2022/AE000853).

## 3 Conclusion

In summary, our findings establish antigen‐detected NMR as a practical framework for minimal epitope engineering and structure‐guided protein binder discovery. Using Na_V_1.7 as a model system, we show that isotope‐labelled minimal antigens can be used both to verify retained local structure and to guide binder selection through solution‐state analysis of binding and epitope engagement. In combination with AlphaFold modelling, this enabled prioritization of binders predicted to target the extracellularly accessible surface of the native channel. Applying this workflow identified R4C8, a subtype‐ and species‐selective nanobody that binds human Na_V_1.7 with minimal effects on channel gating. More broadly, this work highlights antigen‐detected NMR as a useful medium‐throughput strategy for validating and selecting protein binders against minimal, conformationally constrained peptide epitopes.

## Author Contributions

**Junyu Liu**: conceptualization, formal analysis, writing – original draft, investigation, writing – review and editing, visualization, data curation. **Wanlin Chen**: investigation. **Ben Cristofori‐armstrong**: investigation, conceptualization, data curation, formal analysis, visualization, writing – original draft, supervision, project administration, writing – review and editing, methodology. **Theo Crawford**: conceptualization, data curation, formal analysis, visualization, writing – original draft, methodology, investigation, supervision, project administration, writing – review and editing. **Kai‐En Chen**: investigation, formal analysis, visualization. **Peng Xie**: investigation, formal analysis, visualization. **Rainbow Wing Bo Chan**: investigation. **Yifei Zhu**: investigation. **Mimi Golder**: investigation. **Anneka Pereira Schmidt**: investigation. **Jennifer D. Naughton**: investigation. **Nicholas D. Condon**: investigation, visualization, formal analysis. **Åsa Andersson**: investigation. **Farhad Dehkhoda**: investigation. **Kirsten L. Mcmahon**: investigation. **Tabea Klasfauseweh**: investigation. **Ashvriya Thapa**: investigation. **Hue Tran**: investigation. **Poanna Tran**: investigation. **Sina Jami**: investigation. **Lotten Ragnarsson**: investigation. **Sebastian G. B. Furness**: investigation, conceptualization, formal analysis, funding acquisition, resources. **Jennifer R. Deuis**: investigation, formal analysis, visualization, writing – review and editing. **Brett M. Collins**: writing – review and editing, investigation, data curation, formal analysis, visualization, funding acquisition, supervision. **Wai‐Hong Tham**: formal analysis, visualization, writing – original draft, writing – review and editing, supervision, funding acquisition. **Indira Prasadam**: funding acquisition, resources, investigation, supervision, writing – review and editing. **Irina Vetter**: funding acquisition, resources, investigation, writing – original draft, writing – review and editing, supervision. **Mehdi Mobli**: conceptualization, data curation, formal analysis, visualization, writing – original draft, methodology, investigation, supervision, project administration, writing – review and editing, validation, funding acquisition, resources.

## Conflicts of Interest

The authors declare no conflicts of interest.

## Supporting information




**Supporting File**: advs77611‐sup‐0001‐SuppMat.docx.

## Data Availability

The data that support the findings of this study are available in the supplementary material of this article.
