# Supplementary material for: Antigen‐Detected NMR for Minimal Epitope Engineering and Structure‐Guided Selection of a NaV1.7‐Selective Nanobody
Source: Adv Sci (Weinh). 2026 Sep 6:e77611. Online ahead of print. doi: 10.1002/advs.77611 (PMC13547701; doi:10.1002/advs.77611)
Supplement: Supplementary file 1 [file ADVS-9999-e77611-s001.docx]

# **Supporting Information for**

Antigen-Detected NMR for Minimal Epitope Engineering and Structure-Guided Selection of a Na_V_1.7-Selective Nanobody

Junyu Liu^[a,b]^, Wanlin Chen^[b]^, Ben Cristofori-Armstrong^[a]^, Theo Crawford^[a]^, Kai-En Chen^[b]^, Peng Xie^[c,d]^, Rainbow Wing Bo Chan^[e]^, Yifei Zhu^[a]^, Mimi Golder^[a]^, Anneka Pereira Schmidt^[a]^, Jennifer D. Naughton^[a]^, Nicholas D. Condon^[b]^, Åsa Andersson^[b]^, Farhad Dehkhoda^[f]^, Kirsten L. McMahon^[b]^, Tabea Klasfauseweh^[b]^, Ashvriya Thapa^[b]^, Hue Tran^[b]^, Poanna Tran^[b]^, Sina Jami^[b]^, Lotten Ragnarsson^[b]^, Sebastian G. B. Furness^[f,g]^, Jennifer R. Deuis^[b]^, Brett M. Collins^[b]^, Wai-Hong Tham^[e,h,i]^, Indira Prasadam^[c,d]^, Irina Vetter^[b,j]^ & Mehdi Mobli*^[a]^

[a]	Dr. J. Liu, Dr. B. Cristofori-Armstrong, Dr. T. Crawford, Dr. Y. Zhu, M. Golder, A. Pereira Schmidt, J. D. Naughton, Prof. M. Mobli
Australian Institute for Bioengineering and Nanotechnology
The University of Queensland
St Lucia, QLD 4072, Australia
E-mail: [m.mobli@uq.edu.au](mailto:m.mobli@uq.edu.au)

[b]	Dr. J. Liu, Dr. W. Chen, Dr. K.-E. Chen, Dr. N. D. Condon, Å. Andersson, Dr. K. L. McMahon, Dr. T. Klasfauseweh, A. Thapa, Dr. H. Tran, Dr. P. Tran, Dr. S. Jami, Dr. L. Ragnarsson, Dr. J. R. Deuis, Prof. B. M. Collins, Prof. I. Vetter
Institute for Molecular Bioscience
The University of Queensland
St Lucia, QLD 4072, Australia

[c]	P. Xie, A. Prof. I. Prasadam
Centre for Biomedical Technologies, Faculty of Engineering
Queensland University of Technology
Brisbane, QLD 4059, Australia

[d]	P. Xie, A. Prof. I. Prasadam
School of Mechanical, Medical & Process Engineering
Queensland University of Technology
Brisbane, QLD, Australia

[e]	Dr. R. W. B. Chan, Prof. W.-H. Tham
The Walter and Eliza Hall Institute of Medical Research
Parkville, VIC, Australia

[f]	Dr. F. Dehkhoda and A. Prof. S. G. B. Furness
School of Biomedical Sciences
The University of Queensland
St Lucia, QLD 4072, Australia

[g]	A. Prof. S. G. B. Furness
Monash Institute of Pharmaceutical Sciences
Monash University
Melbourne, VIC, Australia

[h]	Prof. W.-H. Tham
Department of Medical Biology
The University of Melbourne
Victoria, Australia

[i]	Prof. W.-H. Tham
Research School of Biology
The Australian National University
Canberra, ACT, Australia

[j]	Prof. I. Vetter
School of Pharmacy and Pharmaceutical Sciences
The University of Queensland
Woolloongabba, QLD, Australia

**Experimental**

*1.1 Production of p-loops*

P-loop 3 was produced recombinantly. A gene encoding p-loop 3 was subcloned into an expression vector (plasmid backbone RRID: Addgene_72668) containing a coding region with poly-histidine purification tag as well as a small ubiquitin-like modifier (SUMO) tag with a tobacco etch virus (TEV) protease cleavage site located between the SUMO tag and p-loop 3. The expression vector was transformed into the BL21(DE3), which were grown in LB media (Miller’s) supplemented with ampicillin at 30 °C until OD_600_ = 0.6-0.8, then induced by adding isopropyl β-D-1-thiogalactopyranoside (IPTG) to a final concentration of 1 mM and incubated for 5 h at 30 °C with shaking. Purification was performed using immobilized metal affinity chromatography (IMAC) and reverse-phase high performance liquid chromatography (RP-HPLC). For IMAC purification, cells were resuspended in buffer (20 mM Tris, 500 mM NaCl, pH 7.8) and lysed using sonication. The soluble cell lysate was applied to a buffer equilibrated 5 mL Ni-NTA column (Cytiva), and the fusion proteins were eluted using buffer containing 250 mM imidazole. Prior to RP-HPLC, cleavage using TEV protease was performed in a mildly reducing redox environment (20 mM Tris, 100 mM NaCl, 0.4 mM oxidized glutathione, 0.6 mM reduced glutathione, pH = 7.8) at room temperature for 3 h. The cleaved p-loop 3 sample was acidified with trifluoroacetic acid (TFA) to a final concentration of 1% and separated via the RP-HPLC consisting of a Shimadzu CBM-20A communication module and Shimadzu LC-20AT pump with Shimadzu SPD-20A detector operating at 214 nm and 280 nm. The mobile phase consisted of solvent A (water with 0.05% TFA) and solvent B (90% acetonitrile with 0.043% TFA). The acidified cleavage product was loaded to a semi-preparative column (Zorbax 300SB-C3, 250 x 9.6 mm, Agilent) operating at a flow rate of 3 ml/min. The elution gradient was increased from 20% B to 60% B over 40 min. The quality control was performed using an analytical column (Zorbax C18, 250 x 4.6 mm, Agilent) operating at a flow rate of 1 ml/min, with a linear elution gradient from 20% B to 60% B over 40 min, and mass validation by matrix-assisted laser desorption/ionisation-time of flight (MALDI-TOF, Bruker Autoflex Speed) mass spectrometry.

Na_V_1.6 & 1.7 p-loop 1 (immunization & display) were synthesized by Liberty Prime automatic synthesizer (CEM, Matthews, North Carolina, US) using Wang-PS-resin. Peptide was cleaved from solid support in a cleavage environment consisting of 92.5% TFA, 2.5% triisopropylsilane, 2.5% H2O, and 2.5% 3,6-dioxa-1,8-octanedithiol for 35 min at 40 °C, when side chains were simultaneously deprotected. After evaporating excess TFA using nitrogen flow, cleaved peptides were precipitated and washed several times using ice-cold diethyl ether, followed by lyophilization for purification. The crude lyophilized peptide was dissolved using a mixture of solvent A and solvent B, then loaded onto a preparative column (Sunfire C18, 19 × 150 mm, Waters), with an elution gradient increasing from 20% B to 60% B over 40 min. An additional purification was performed using a semi-prep column (Zorbax 300SB-C3, 250 x 9.6 mm, Agilent), with the same elution gradient. The quality of purified peptides was validated with a C18 column (Jupiter C18, 250 x 4.6 mm, Phenomenex) and MALDI-TOF (Bruker Autoflex Speed) mass spectrometry as described above. P-loop 1 containing azido-lysine was biotinylated through click chemistry. 1 mM purified p-loop 1, 2 mM biotin-PEG4-alkyne, 3 mM CuSO_4_, 3.5 mM THPTA, 30 mM aminoguanidine, and 30 mM sodium ascorbate were dissolved in phosphate buffer (pH 7.0) with nitrogen flow protection. The reactions were incubated at 37 °C for one hour with gently shaking and stopped by adding TFA to 1%. The reaction products were purified by RP-HPLC with a C18 column (Jupiter C18, 250 x 4.6 mm, Phenomenex). The quality of biotinylated p-loop 1 was validated using the same column and MALDI-TOF (Bruker Autoflex Speed) mass spectrometry.

Isotopically labelled p-loop 1 (epitope mapping) was produced recombinantly. A gene encoding p-loop 1 was subcloned into an expression vector containing a coding region with poly-histidine purification tag and SUMO tag. The expression vector was transformed into the BL21(DE3), which were grown in minimal media (3 g/L KH_2_PO_4_, 12.8 g/L Na_2_HPO_4_, 1 g/L NaCl, 2 mM MgSO_4_, 0.1 mM CaCl_2_, 1x BME vitamin mix (Sigma Aldrich), 1 g/L ^15^NH_4_Cl, 4 g/L ^13^C glucose). The induction, extraction, cleavage (using SUMO protease), and purification were performed in a similar manner to p-loop 3.

*1.2 Serum stability assay*

Mouse serum and diluted 1:1 with DPBS, then pre-incubated at 37 °C for 15 minutes to activate endogenous proteases. P-loop stock was added to the diluted serum to a final working concentration of 25 μM. Samples were incubated at 37 °C, and 5 μL aliquots were collected at 0, 15, 30, 45 minutes, and 1, 2, 4, and 6 hours. Each aliquot was immediately quenched by mixing with 5 μL of 8 M urea and incubating on ice for 10 minutes, followed by the addition of 0.2 μL of 50% TCA and further incubation at 4 °C for 10 minutes. Samples were then centrifuged at 14,000 *g* for 10 minutes at room temperature, and the supernatant was diluted with deionized (Milli-Q) water to a final peptide concentration of 1 μM. 1 μL volume of the diluted supernatant was injected onto an analytical RP-HPLC column (Luna Omega C18, 50 × 2 mm, Phenomenex) and detected using a Bruker TIMS TOF Flex mass spectrometer. Extracted ion chromatograms were generated using an m/z window of M^3^⁺ ± 0.01. Peak areas were integrated using the Integration Method in Bruker DataAnalysis software (v6.1). Peak areas were extracted and analyzed in GraphPad Prism (v10.5.0) with simple linear regression.

*1.3 Immunization, nanobody library construction, and bio-panning*

One alpaca each was immunized six times with approximately 160 μg of p-loop 1 or p-loop 3 on days 0, 14, 21, 28, 35, and 42. One alpaca was immunized with each antigen using GERBU Adjuvant FAMA (GERBU Biotechnik GmbH, Heidelberg, Germany), a particulate adjuvant formulated for sensitive large animals, according to the established protocol. All procedures involving the immunization and handling of alpacas were approved by the Agriculture Victoria Wildlife & Small Institutions Animal Ethics Committee (project approval No. 36.20). Blood was collected three days after the final immunization for lymphocyte preparation. Subsequent library construction and phage display panning was performed by WEHI Nanobody Platform.

Nanobody library construction was performed following established protocols.^[1]^ Briefly, mRNA was extracted from alpaca lymphocytes and amplified via RT-PCR using specific primers to generate a cDNA library encoding nanobody sequences. The nanobody library was cloned into the pMES4 phagemid vector, amplified in E. coli TG1 cells, and subsequently infected with M13K07 helper phage to enable recombinant phage expression.

Phage display was performed using both the immunized nanobody library and proprietary library. The proprietary library has 10^5^ unique nanobody clones. Two bio-panning strategies were employed: one with antigens immobilized on neutravidin-coated plates and the other with antigens immobilized on streptavidin-coated magnetic beads. For the neutravidin-coated approach, 5 ng/μL of neutravidin and 1 μg of biotinylated p-loop 1 were used to coat plates, followed by three rounds of bio-panning. In the streptavidin-coated magnetic bead approach, beads were coated with 500 nM biotinylated p-loop 1, and four rounds of bio-panning were completed.

From both panning campaigns, a total of 188 clones were selected, of which 65% were confirmed as positive hits through ELISA. Positive clones were sequenced, annotated using the International ImMunoGeneTics database, and aligned in Geneious Biologics software. Based on at least one amino acid difference in the CDR3, 14 distinct nanobody clonal groups were identified. Notably, some sequences were found to be common across the two panning conditions.

*1.4 Nanobody production*

Nanobodies were produced through recombinant expression according to established protocol.^[1]^ The plasmid containing gene coding nanobody with a His_6_ tag in its C terminus was transformed into E. coli WK6 cells, which were grown in TB media (20 g/L tryptone, 24 g/L yeast extract, 9.4 g/L K_2_HPO_4_, 2.2 g/L KH_2_PO_4_, 4 ml/L glycerol, 1 g/L glucose, 2 mM MgCl_2_) supplemented with ampicillin at 37 °C with shaking until OD_600_ reached 1.5, then induced by adding IPTG to a final concentration of 1 mM and incubated overnight at 30 °C with shaking. Expressed nanobodies were extracted from periplasm by osmotic shock and purified using IMAC. Depending on the isoelectric point of the nanobody, 40 mM Tris (pH 7.8) or 40 mM HEPES (pH 7.0) was used in the following purification. Cells were harvested by centrifugation at 7,000 *g* for 10 minutes, then resuspended in buffer containing 150 mM NaCl, 40% sucrose (w/w), and 0.5 mM EDTA, followed by incubation on ice for two hours. The cells were subsequently pelleted and resuspended in the ice-cold buffer containing 2 mM MgCl_2_ and incubated on ice for one hour to disrupt the periplasmic membrane. The soluble fraction was applied to a 5 ml IMAC column pre-equilibrated with buffer containing 150 mM NaCl and 10 mM imidazole. Nanobodies were eluted using buffer containing 250 mM imidazole.

Synthetic codon optimized genes for R4C8, R4C8-Sor, and R4C8-HA were purchased (Integrated DNA technologies, Singapore) and subcloned into a modified pMCSG7 expression vector containing T7 promoter and a coding region with a PelB signal peptide. The plasmids were transformed into E. coli BL21(DE3) cells, which were grown in autoinduction media (100 µg/ml ampicillin, 10 g/L tryptone, 5 g/L yeast extract, 25 mM Na_2_HPO_4_, 25 mM KH_2_PO_4_, 50 mM NH_4_Cl, 5 mM Na_2_SO_4_, 2 mM MgSO_4_, 0.5% glycerol, 0.05% glucose, 0.2% α-lactose) supplemented with ampicillin at 30 °C overnight. Cells were harvested by centrifugation and resuspended using denaturing buffer (20 mM Tris, 150 mM NaCl, 8 M urea, pH 8.0), followed by sonication. Supernatants were applied to 5 ml IMAC column (HisTrap FF, Cytiva) pre-equilibrated with denaturing buffer. The nanobodies were eluted using denaturing buffer containing 250 mM imidazole and refolded via dialysis into urea-free buffer (20 mM Tris, 150 mM NaCl, pH 8.0) using a dialysis bag with a cut-off of 14 kDa.

R4C8-FITC was produced using a R4C8-Sor construct containing a C-terminal LPGTG sortase recognition sequence prior to a histidine affinity tag. Purified R4C8-Sor (25 µM) was incubated with GGGK-FITC (75 µM) and Sortase A (5 µM) in 50 mM Tris, pH 8.0, 50 mM NaCl, 1 mM CaCl_2_, and 10% glycerol overnight at room temperature in the dark. The reaction was applied to a Ni^2+^-IMAC column. His-tagged R4C8-Sor and Sortase A were retained, whereas ligated R4C8-FITC, which no longer contained the C-terminal His tag, was recovered in the flow-through and wash fractions. Product identity and purity were confirmed by SDS-PAGE, fluorescence imaging, and mass spectrometry.

*1.5 CD and NMR characterization*

P-loops were dissolved in 10 mM sodium phosphate buffer, pH 7.4, at 20 μM and loaded in 1-mm path-length quartz cuvettes for scanning. CD spectra were recorded at room temperature (298 K) over wavelengths ranging from 185 to 260 nm in 0.5-nm intervals and sensitivity of 20-50 mdeg using a Jasco J-810 CD spectropolarimeter. Each spectrum represents the average of five scans. Spectra were analyzed using the spectral analysis software and smoothed using ‘adaptive smoothing’ function.

The resonance assignment of p-loop 1 was conducted using heteronuclear NMR. The sample contained 100 μM ^13^C/^15^N-labeled p-loop 1 in 20 mM phosphate buffer pH 7.4, 10 μM sodium trimethylsilylpropanesulfonate, 5% D_2_O, 1 mM EDTA and 0.02% NaN_3_. All spectra were recorded at 25 °C on a Bruker 700 MHz spectrometer equipped with a triple resonance cryogenically cooled probe (Bruker, Billerica, Massachusetts, USA), unless otherwise stated. Resonance assignments were obtained from two-dimensional (2D) ^1^H/^15^N HSQC, three-dimensional (3D) HNCACB, 3D CBCA(CO)NH, and 3D HNCO. 3D spectra were collected using non-uniform sampling and processed using automated maximum entropy reconstruction.^[2]^ The secondary structure of p-loop 1 was predicted based on chemical shifts using TALOS-N.^[3]^

^1^H/^15^N HSQC spectra of p-loop 1 with and without the presence of nanobody were acquired. Each sample contained 100 μM ^15^N-labelled p-loop 1 in 20 mM phosphate buffer, pH 7.4, 5% D_2_O, either with 50 μM nanobody, 200 μM nanobody, or in the absence of nanobody.

zz-exchange spectra of p-loop 1 in the presence of nanobody were recorded at 30 °C. Each sample contained 100 μM ^15^N-labelled p-loop 1 and 50 μM nanobody in 20 mM phosphate buffer, pH 7.4, 5% D_2_O. A ^1^H/^15^N HSQC spectrum was recorded under the same condition. All NMR spectra were processed using Topspin (v3.6.1, Bruker), and analyzed using CCPNMR (v2.4.2).^[4]^ The chemical shift changes were calculated using the following equation:

$\Delta\delta= \sqrt{\left( \delta_{H}- \delta_{H}^{'} \right)^{2}+\left[ \frac{1}{10}\left( \delta_{N}- \delta_{N}^{'} \right) \right]^{2}}$

$\Delta\delta$ refers to the chemical shift perturbation of a peak. $\delta_{H}$ and $\delta_{N}$ refer to the chemical shift of the peak in proton and ^15^N in unbound state. $\delta_{H}^{'}$ and $\delta_{N}^{'}$ refer to the chemical shift in proton and ^15^N of the peak in bound state.

*1.6 AlphaFold prediction & multiple sequence alignment*

AlphaFold v2.3.2 was installed locally and employed for the prediction of nanobody-p-loop 1 complex, using its proprietary database (updated in March 2023) and “multimer” option. The ipTM value and PAE matrix were provided to each prediction by AlphaFold. The average PAE was calculated by taking the mean of all inter-chain PAE values between the nanobody and the residues in p-loop 1.

*1.7 Isothermal titration calorimetry (ITC)*

All microcalorimetry experiments were carried out by titrating 200 μM nanobody into 20 μM p-loop 1 at 25˚C using a PEAQ ITC (Malvern, UK) in 20 mM phosphate buffer, pH 7.4. Each experiment was performed with a single injection of 0.4 μL followed by a series of 12 injections of 3.2 μL each with intervals of 180 seconds and stirring of 850 rpm. The heat exchange of interactions was calculated by integrating the observed peaks and subtracting the heats of dilution from the background. The thermodynamic parameters for all binding tests were obtained by fitting and normalizing data to a single-site binding model using the Malvern software package. To ensure the data were reproducible, each experiment was run in triplicate.

*1.8 Crystallization and Data Collection*

For co-crystallization, 1.2-fold molar excess of the disulfide bond stabilized p-loop 1 fragment was added to the purified nanobody R3D8 to a final protein concentration of 20 mg/ml. Crystallization was setup by the hanging drop vapor diffusion method at 20 °C. Initial crystallization screen conducted at neutral pH buffer (50 mM Tris-HCl pH 7.5) was unsuccessful. Crystals were observed in multiple different commercial screen condition after changing both R3D8 and p-loop 1 fragment to 100 mM ammonium acetate pH 5.5 buffer. Best diffracting quality crystals was observed after 2 days under the condition comprising 50 mM sodium cacodylate pH 6.5, 90 mM ammonium sulfate, 13% PEG 8000. Prior to the X-ray data collection, crystals were cryoprotected in crystallization solution mentioned above with the addition of 10% glycerol. X-ray diffraction data was collected at the Australian Synchrotron MX2 beamline at 100K.

1.9 Crystal structure Determination

Data collected from the Australian Synchrotron were indexed and integrated by AutoXDS and scaled using Aimless (version 0.7.4).^[5]^ The structure was solved by molecular replacement using Phaser (version 2.5.6)^[6]^ with AlphaFold model as the initial template. The structure contains one copy of nanobody in asymmetric unit with the electron density of the P-loop1 clearly visible. The refinement was performed using Phenix (version 1.19.2)^[7]^ with inspection of resulting model in Coot (version 0.8.9.2) guided by the electron density maps. Molprobity was used to evaluate the geometric quality of the refined structure.^[8]^ Data collection and refinement statistics are summarized in Supplementary Table S3. Molecular figures were generated using PyMOL.

1.10 Immunofluorescence

Human Embryonic Kidney (HEK) 293 cells (RRID: CVCL_0045) stably expressing Na_V_1.1/β1 – Na_V_1.7/β1 (SB Drug Discovery, Glasgow, United Kingdom) and untransfected HEK293 cells were maintained in MEM supplemented with 10% (v/v) FBS, L-glutamine (2 mM), and selection antibiotics. The selection antibiotics included geneticin (600 µg/mL, Sigma-Aldrich, Missouri, USA) and blasticidin (4 µg/mL, Thermofisher, Massachusetts, USA) for all cell lines, with additional zeocin (500 µg/mL, ThermoFisher, Massachusetts, USA) for HEK293-Na_V_1.4 cells. Chinese Hamster Ovary (CHO) cells (RRID: CVCL_0213) stably expressing tetracycline-inducible hNa_V_1.8/β3 (ChanTest, Ohio, USA) were cultured in MEM additionally supplemented with 10% (v/v) FBS and 2 mM L-glutamine. Tetracycline (1 µg/mL, Sigma-Aldrich, Missouri, USA) was freshly prepared from frozen stocks and added 48 h prior to functional assays to induce Na_V_1.8 expression. HEK293 expressing Ms Na_V_1.7 were cultured according to protocol described in previous study.^[9]^

All cell lines were cultured at 37 °C in a humidified incubator with 5% CO₂ and passaged using TrypLE Express (ThermoFisher, Massachusetts, USA).

Cells as above were seeded in 6-well plates onto 18 mm diameter No. 1/1.5 square coverslips several days before the experiment to achieve approximately 70% confluency at the time of treatment. On the day of the experiment, coverslips were washed by immersion in Dulbecco's Phosphate-Buffered Saline (DPBS, ThermoFisher, Massachusetts, USA) and fixed with Electron Microscopy grade 4% paraformaldehyde in DPBS (#C004, ProSciTech) for 30 minutes at room temperature. After fixation, the cells were washed thoroughly with DPBS. Fixed cells were not permeabilized.

Fixed cells were incubated with 10 µM R4C8-HA diluted in DPBS containing 0.5% (w/v) bovine serum albumin (BSA, Merck, Massachusetts, USA), rabbit anti-HA antibody (RRID: AB_260070, #H6908, Merck, Massachusetts, USA, 1:1000 dilution in DPBS containing 0.5% BSA), and anti-rabbit secondary antibody conjugated to Atto-647 fluorescent dye (RRID: AB_2752244, #A150075, Abcam, Cambridge, United Kingdom, 1:1000 dilution in DPBS containing 0.5% BSA) for 1 hour, respectively, with a wash between each step.

For R4C8-FITC labelling, fixed cells were incubated with 1 µM R4C8-FITC diluted in DPBS containing 0.5% bovine serum albumin overnight.

For colocalization experiments, fixed cells were first incubated with 10 µM R4C8-HA dissolved in DPBS containing 0.5% BSA for 1 hour and washed. Cells were then incubated in DPBS containing 0.5% BSA and a 1:1000 dilution of both rabbit anti-Na_V_1.7 antibody (RRID: AB_2341069, #ASC-027, Alomone Labs) and mouse anti-HA antibody (RRID: AB_260092, #H9658, Merck, Massachusetts, USA). ASC-027 was raised against residues 1533–1543 of rodent Na_V_1.7, corresponding to the extracellular S1–S2 loop of voltage-sensing domain IV, which is spatially distinct from p-loop 1. The product is reported to cross-react with human Na_V_1.7 by the manufacturer. After washing, cells were incubated with donkey anti-mouse secondary antibody conjugated to Atto-647 fluorescent dye (RRID: AB_162542, #A31571, Abcam, Cambridge, United Kingdom) and donkey anti-rabbit secondary antibody conjugated to Atto-488 fluorescent dye (RRID: AB_2636877, #150073, Abcam, Cambridge, United Kingdom), each diluted 1:1000 in DPBS supplemented with 0.5% BSA. Cells were washed thoroughly after incubation.

The coverslips were mounted using 4′,6-diamidino-2-phenylindole (DAPI)-containing mounting medium (Abcam, Cambridge, United Kingdom) and imaged using a 63x 1.4NA plan apochromat oil immersion objective or a 100x 1.4NA A-plan apochromat oil immersion objective on Zeiss Axiovert 200 Inverted Microscope Stand using LSM 710 Meta NLO Confocal Scanner with relevant lasers and detector gating running Zen Black.

1.11 Whole-cell patch-clamp

Automated whole-cell patch-clamp experiments were conducted using a QPatch II electrophysiology platform (Sophion Bioscience, Ballerup, Denmark). Cells at 70–80% confluency were harvested from T75 flasks using TrypLE Express at 37 °C for 2–5 minutes and resuspended in DMEM supplemented with 25 mM HEPES (Sigma-Aldrich, NSW, Australia), 100 U/mL penicillin-streptomycin (Gibco), and 40 µg/mL trypsin inhibitor from Glycine max (soybean) (Sigma-Aldrich). The resuspended cells were allowed to recover on a QStirrer (Sophion) for 30 minutes before the experiment.

Automated patch-clamp recordings were performed using 16-channel patch plates with a patch hole diameter of 1 µm and resistance of 2 ± 0.02 MΩ. Cell positioning and sealing parameters were set as follows: positioning pressure of −60 mbar, minimum seal resistance of 0.1 GΩ, holding potential of −100 mV, and holding pressure of −20 mbar. Leak currents and capacitive transients were minimised using online P/4 leak subtraction.

Manual patch-clamp recordings were used for deactivation data, cells were passaged as above and plated onto coverslips coated with 0.1% Poly-L-lysine. Recordings were performed at room temperature (~23 °C) using a Multiclamp 700B amplifier and digitized with a Digidata 1550B and pClamp 10.7 software (Molecular Devices). Giga seals were formed using patch pipettes pulled from borosilicate glass to a final tip resistance of 2–6 MΩ, and data were collected from cells with access resistance <10 MΩ. Leak currents and capacitive transients were minimized using online P/2 leak subtraction.

All whole-cell currents were filtered at 5 kHz and acquired at 25 kHz, with series resistance compensation set to 70%.

The extracellular solution (ECS) contained the following components (in mM): 75 mM choline chloride, 75 NaCl, 4 KCl, 2 CaCl₂, 1 MgCl₂, 10 HEPES, and 10 glucose, adjusted to pH 7.4 and an osmolarity of 305 mOsm. The intracellular solution consisted of (in mM): 140 CsF, 1/5 EGTA/CsOH, 10 HEPES, and 10 NaCl, adjusted to pH 7.3 with CsOH and an osmolarity of 320 mOsm.

Representative currents from the cell lines used in the immunofluorescence selectivity panel were recorded using the same automated whole-cell patch-clamp configuration. Untransfected HEK293 cells, HEK293 cells stably expressing human Na_V_1.1/β1-Na_V_1.7/β1, and CHO cells stably expressing human Na_V_1.8/β3 were held at −90 mV and subjected to a single depolarising voltage step to −20 mV, except for Na_V_1.8/β3 cells, which were stepped to +10 mV.

Current-voltage (I−V) relationships were determined in the absence of R4C8 by applying a series of 500 ms step pulses from −110 to +80 mV in 5 mV increments, with a 5-second interval between pulses, from a holding potential of −90 mV. After incubation with R4C8 (10 µM), the same protocol was repeated. Whole-cell currents were converted to conductance (G) using the formula G = I/(V − V_rev_), where the reversal potential (V_rev_) is set +57.5 mV. Conductance was plotted against voltage, and conductance-voltage (G-V) curves were fitted using a Boltzmann equation to retrieve V50 of activation values. Current inactivation time constants were analyzed from activation curves by fitting a standard exponential function, with data plotted for currents recorded between −30 mV and +40 mV.

The voltage dependence of steady-state fast inactivation was assessed by applying a 10 ms pulse to −20 mV immediately following the 500 ms depolarisation steps described above, to determine the fraction of non-inactivated channels. The data was fitted to a Boltzmann equation and V_50_ of inactivation values were plotted for statistical comparison.

The deactivation kinetics protocol consisted of a conditioning period at −120 mV followed by a 0.5 ms voltage step to −10 mV and a series of 50 ms pulses at a range of potentials from −120 to −50 mV with 10 mV increments (sweeps every 12 s). The decay time (from peak to 20% peak amplitude) of tail current decay was then plotted as a function of the test pulse.

All data are presented as mean ± S.E.M. unless otherwise stated. n values (independent experiments or biological replicates) and statistical tests are detailed in the respective figure legends. Data were plotted and analyzed using GraphPad Prism version 10.1.2. Statistical significance was defined as p < 0.05.

1.12 Two-electrode voltage-clamp

All experiments involving Xenopus laevis frogs were conducted in accordance with relevant national and international regulations, including the Australian Code of Practice for the Care and Use of Animals for Scientific Purposes (8th edition, 2013). Oocyte collection procedures were reviewed and approved by the Anatomical Biosciences group of the Animal Ethics Committee at The University of Queensland (2022/AE000853) and carried out in compliance with Australian quarantine regulations.

The human Na_V_1.7 gene, cloned into a pcDNA3.1 plasmid, was linearised with the NotI restriction enzyme and used as a template for cRNA synthesis with T7 polymerase (mMessage mMachine Kit; Life Technologies). Stage V/VI oocytes were injected with 12 ng of total RNA and incubated at 17 °C for 2–4 days in 50% Leibovitz’s L-15 medium (Gibco), supplemented with 25 µg/ml gentamicin, 25 µg/ml streptomycin, and 2.5% foetal horse serum.

Electrophysiological recordings were performed at room temperature using two-electrode voltage clamp (Axoclamp 900A amplifier; Axon Instruments) in ND96 solution (in mM: 96 NaCl, 2 KCl, 1 MgCl₂, 1.8 CaCl₂, 5 HEPES; pH 7.4 with NaOH), supplemented with 0.01% fatty acid-free bovine serum albumin, in a ~30 μL recording chamber. Borosilicate glass microelectrodes had resistances of 0.2–0.8 MΩ when backfilled with 3 M KCl. Data were digitised at 10 kHz and filtered at 2 kHz using pCLAMP 11 software (Digidata 1550B; Axon Instruments).

R4C8 activity was assessed via constant perfusion of a 10 µM solution for 5 minutes, with currents measured during a 50 ms step to −10 mV every 10 s from a holding voltage of −80 mV. For all other voltage protocols, oocytes were held at −90 mV. Activation curves were obtained by measuring peak currents during steps from −100 mV to +50 mV in +10 mV increments applied every 10 s, with G/G_max_ plots spanning −80 mV to +10 mV. Current inactivation time constants were analyzed from activation curves by fitting a standard exponential function, with data plotted for currents recorded between −30 mV and +40 mV. Steady-state inactivation curves were generated by plotting the peak current at a test pulse of −10 mV, following a 200 ms pre-pulse from −100 mV to +50 mV in +10 mV increments applied every 10 s.

All data are presented as mean ± S.E.M. unless otherwise stated. n values (independent experiments or biological replicates) and statistical tests are detailed in the respective figure legends. Data were plotted and analyzed using GraphPad Prism version 10.1.2. Statistical significance was defined as p < 0.01.

1.13 Chondrocytes

Femoral cartilage was obtained from patients undergoing primary total knee arthroplasty surgery. The ethics committees of Queensland University of Technology and St Vincent Private Hospital approved this study, and all participants provided informed consent (ethics number: #1400001024). The cartilage was minced into small pieces and washed three to four times with phosphate-buffered saline. Chondrocytes were isolated by digestion with 1% type II collagenase (Gibco, Thermo Fisher Scientific) for 6 to 10 hours and resuspended in low-glucose Dulbecco's modified Eagle's medium (DMEM; Gibco) supplemented with 10% fetal bovine serum (FBS; Gibco) and 1% penicillin-streptomycin (Gibco) (complete medium) as previously described^[10]^.

Cells were labelled and mounted in the same way as described above. Slides were imaged using a 60x objective in Olympus Evident FV4000 confocal microscopy (R4C8-FITC, Pn3a) or using a 20x objective in Olympus BX51 immunofluorescent microscopy (R4C8-HA).

1.14 Flow cytometric cell-binding assessment

HEK293 cells stably expressing human Na_V_1.7/β1 or β1/β2 were maintained as described above. Dissociated cells in DPBS containing 1% FBS were aliquoted into a 96-well plate at 4 x 106 cells/well and incubated with 0 (non-treated), 0.05, 0.1, 0.5, 1, 5, or 10 µM R4C8-FITC for 30 minutes at room temperature. Cells were collected by centrifugation (5 min, 200 xg) and gently resuspended in DPBS containing 1% FBS, repeated twice. A blocking experiment was performed by first incubating cells with 10 µM R4C8 for 30 minutes, removing the solution and then incubating with 1 µM R4C8-FITC. Samples were prepared in triplicate and analysed using a Cytoflex S flow cytometer (Beckman Coulter, USA), data was processed using CytExpert version 2.4.0.28 (Beckman Coulter, USA) to obtain mean fluorescence intensity values and robust standard deviations. Data was plotted using GraphPad Prism version 11.0.0.

For transient transfection, FreeStyle 293-F cells (Gibco, Thermo Fisher Scientific, cat. no. R79007) were maintained in FreeStyle 293 Expression Medium (Gibco, Thermo Fisher Scientific, cat. no. 12338018) at 37°C in a humidified atmosphere containing 8% CO₂ with agitation. Cells were seeded at 1 × 10⁶ cells/ml, and 1 × 10^7^ cells were used for transfection. Cells were either left untransfected or transiently transfected with 20 µg of Na_V_1.7-IRES-GFP (plasmid backbone RRID: Addgene_80344) expression plasmid. Plasmid DNA was diluted in Opti-MEM and complexed with PEI MAX (Polysciences, cat. no. 24765) at a DNA:PEI mass ratio of 1:4, corresponding to 20 µg DNA and 80 µg PEI MAX. Transfection complexes were incubated at room temperature for 30 minutes before being added to the cells, which were then incubated for 24 hours. Cells were subsequently collected by centrifugation and washed twice with HBSS binding buffer comprising Hank’s Balanced Salt Solution supplemented with 0.05% ovalbumin, 20 mM HEPES, and 0.05% sodium azide (centrifugation steps were performed at 4°C for 3 min at 400 ×g). Following washing, control and transfected cells were resuspended in binding buffer and divided equally among 4 wells of a V-bottom 96-well plate. For fluorescent nanobody preparation, one well was left unstained, while the remaining three wells were incubated with 10, 100, or 1,000 nM Cy5-conjugated R4C8 nanobody in a final volume of 200 µl of binding buffer. Cells were incubated for 4 hours at 4°C in the dark. Following incubation, cells were centrifuged but not washed and maintained in the nanobody-containing buffer until immediately before analysis. The supernatant was then carefully aspirated, and cells were resuspended in 200 µl of binding buffer containing 500 nM SYTOX Blue. Samples were analysed using a BD Fortessa flow cytometer, and raw data were processed using FlowJo (Becton, Dickinson and Company, version 10.6.2) to identify single, live cells. Mean fluorescence intensity (MFI) values were then obtained for each sample and compared across the unstained, 10, 100, and 1,000 nM conditions for untransfected and Na_V_1.7-IRES-GFP-transfected cells.

**Figure S1.** Integrated “Antigen-Detected NMR” workflow for minimal antigen engineering and nanobody selection. The seven-step pipeline encompasses epitope selection, antigen engineering and qualification, nanobody discovery and triage, interface mapping, and cellular validation, culminating in identification of R4C8 as a selective probe for human Na_V_1.7.

**Figure S2.** Sequence alignment and AlphaFold2 prediction used in epitope engineering of p-loops. (a) Multiple sequence alignment of p-loop 1 from human Na_V_ isoforms generated using Clustal Omega,^[11]^ with conservation scores shown as bar chart.^[12]^ The Na_V_1.7-specific regions are highlighted in magenta. Residues are numbered based on Na_V_1.7 (UniProt ID: Q15858). (b) Multiple sequence alignment of p-loop 3 from human Na_V_ isoforms generated using Clustal Omega, with conservation scores shown as bar chart. The Na_V_1.7-specific regions are highlighted in cyan. Residues are numbered based on Na_V_1.7 (UniProt ID: Q15858). (c) Side view of human Na_V_1.7 (PDB ID: 6J8G) with extracellular regions of pore domain I (right) and III (left) highlighted. (d-e) Sequence of extracellular region of pore domain III (d) and p-loop 1 (e). The disulfide bond is indicated in orange, and the artificial cysteine is highlighted by a red arrow. (f) Superimposition of extracellular region in pore domain III (grey) and the isolated p-loop 3 (cyan, predicted by AlphaFold2), with a backbone RMSD = 0.9 Å. The native p-loop 3 disulfide bond is shown in orange sticks. (g) Superimposition of extracellular region in pore domain I (grey) and the isolated p-loop 1 (magenta, predicted by AlphaFold2), with a backbone RMSD = 2.3 Å. The engineered disulfide bond is shown in orange sticks, and the artificial cysteine is highlighted by a red arrow.^[13]^ The cysteine residue (C275) is shown in orange. (h) Predicted structure of native p-loop 1 in isolation using AlphaFold2.^[13]^ The sidechain of the native cysteine residue (C275) and the mutated tyrosine shown (Y304) as sticks.

**Figure S3.** Production, and NMR characterization of p-loops. (a) HPLC chromatogram and MALDI-TOF mass spectrum of p-loop 1 (top) and p-loop 3 (bottom) used for immunization & display (expected mass of p-loop 1 3750; expected mass of p-loop 3 4070). (b) Circular dichroism (CD) spectra of p-loop 1 (top, magenta) and p-loop 3 (bottom, cyan) under reducing (dotted) and non-reducing (solid) conditions. All spectra were recorded at a concentration of 20 μM in 10 mM phosphate buffer, pH 7.4. (c) ^1^H-^15^N HSQC spectrum of 100 µM ^15^N-labelled p-loop 1 with backbone amide assignments.

**Figure S4.** P-loop 1 immunization and nanobody diversity. (a) Sequence (left), chromatogram (middle), and mass spectrum (right, expected mass 4648) of biotinylated Na_V_1.7 p-loop 1. Disulfide bond is indicated in orange line. (b) Sequence (left), chromatogram (middle), and mass spectrum (right, expected mass 4502) of Na_V_1.6 p-loop 1. Disulfide bond is indicated by an orange line. (c) ELISA results of phage clones enriched from immunization-induced and proprietary libraries against p-loop 1. Clones with absorbance values above the threshold (dashed line) were considered positive binders. Binding was detected using anti-M13 HRP-conjugated antibody, and absorbance was measured at 405 nm. (d) Multiple sequence alignment of CDR regions in nanobodies, generated using T-Coffee ^[11]^, with conservation scores shown as bar chart.

**Figure S5.** Binding of p-loop 1 with R3D8, R4A7, R4C8, and R4F12, assessed by NMR. Overlay of ^1^H-^15^N HSQC spectra of 100 µM ^15^N-labelled p-loop 1 before (red) and after adding 50 µM (blue) or 200 µM of nanobodies (green).

**Figure S6.** Binding of p-loop 1 with R3D3, R3H9, R4B9, and R4D3, assessed by NMR. Overlay of ^1^H-^15^N HSQC spectra of 100 µM ^15^N-labelled p-loop 1 before (red) and after adding 50 µM (blue) or 200 µM of nanobodies (green).

**Figure S7.** Binding of p-loop 1 with R4C9, R4G10, R4E9, and R3F8, assessed by NMR. Overlay of ^1^H-^15^N HSQC spectra of 100 µM ^15^N-labelled p-loop 1 before (red) and after adding 50 µM (blue) or 200 µM of nanobodies (green).

**Figure S8.** Isothermal titration calorimetry (ITC) data for nanobodies R3D8 (blue), R4A7 (orange), R4C8 (green), and R4F12 (purple) titrated into p-loop 1. Left, differential heat plots for the different titrations are offset for clarity. Right, a table summarising the fitted ITC parameters.

**Figure S9.** Binding interface mapping of R4C8-p-loop 1 and R3D8-p-loop 1 complexes. (a-b) Predicted binding interface between R4C8 and p-loop 1 based on the top-ranked AlphaFold2 model. (c) Overlay of HSQC (p-loop 1, red) and zz-exchange spectrum (blue, p-loop 1 in the presence of 50 µM R3D8). Dashed lines highlight bound and unbound states of key residues. (d – f) AlphaFold2-predicted interactions between p-loop 1 key residues and R3D8.

**Figure S10.** Binding interface mapping of R4A7-p-loop 1 and R4F12-p-loop 1 complexes. (a) Overlay of HSQC (p-loop 1, red) and zz-exchange spectrum (orange, p-loop 1 in the presence of 50 µM R4A7). Dashed lines highlight bound and unbound states of key residues. (b) Chemical shift perturbations mapped across p-loop 1 residues from the R4A7 zz-exchange experiment. Residues with the chemical shift perturbations larger than threshold (dashed line) are labelled. F31 is inaccessible in native full-length channel and labelled. (c–e) Predicted binding interface between R4A7 and p-loop 1 based on the top-ranked AlphaFold2 model. The backbone amide of S16 (p-loop 1) is predicted to interact with the backbone carbonyl of G99 (R4A7). The side chain of E22 (p-loop 1) is predicted to form a salt bridge with K53 (R4A7). Residues L21 and F31 (p-loop 1), and V33 and A50 (R4A7) contribute to a predicted hydrophobic core. (f) Overlay of HSQC (p-loop 1, red) and zz-exchange spectrum (purple, p-loop 1 in the presence of 50 µM R4F12). Dashed lines highlight bound and unbound states of key residues. (g) Chemical shift perturbations mapped across p-loop 1 residues from the R4F12 zz-exchange experiment. Residues with the chemical shift perturbations larger than threshold (dashed line) are labelled. M18 is inaccessible in native full-length channel and labelled. (h–i) Predicted binding interface between R4F12 and p-loop 1 based on the top-ranked AlphaFold2 model. Residue M18 (p-loop 1) forms a predicted hydrophobic core with A33 and A50 (R4F12). The backbone carbonyls of T20 and L21 (p-loop 1) are predicted to form hydrogen bonds with the side chain of K59 (R4F12).

**Figure S11.** Representative currents from Na_V_ subtype-expressing cell lines used in the R4C8 selectivity panel. Representative whole-cell current traces recorded by automated patch clamp using a QPatch II platform from control HEK293 cells, HEK293 cells stably expressing human Na_V_1.1/β1-Na_V_1.7/β1, and CHO cells expressing human Na_V_1.8/β3. Currents were evoked by a single depolarising voltage step from −90 mV to −20 mV, except for Na_V_1.8, where currents were evoked by a step to +10 mV. Control HEK293 cells showed no detectable Na_V_-like inward current, whereas each Na_V_ subtype-expressing cell line displayed rapidly activating inward currents with subtype-consistent properties. Scale bars are indicated in each panel.

**Figure S12.** Production and purification of R4C8-FITC. (a) Scheme of Sortase A-catalyzed ligation between R4C8 carrying a C-terminal sortase tag (R4C8-Sor) and a GGGK peptide with a FITC-labelled lysine (shown in magenta). The cleavage site is highlighted with black triangle. (b) SDS-PAGE analysis of the ligation reaction and subsequent purification using immobilized metal affinity chromatography (IMAC). The gel was first imaged for FITC fluorescence (left), then stained with Coomassie Blue (right). Fractions contain R4C8-FITC are highlighted with a magenta box. (c) Mass spectrum of fractions containing pure R4C8-FITC (expected mass 13821).

**Figure S13.** Live-cell flow-cytometry assessment of R4C8 binding to full-length Na_V_1.7. (a) Representative histogram of R4C8-FITC fluorescence comparing HEK293-Na_V_1.7/β1 and HEK293-β1/β2 cells stained with 0.5 µM R4C8-FITC. (b) Mean fluorescence intensity (MFI) of HEK293-Na_V_1.7/β1 and HEK293-β1/β2 cells stained with the indicated concentrations of R4C8-FITC. The block condition represents cells pre-incubated with 10 µM unlabelled R4C8 followed by 1 µM R4C8-FITC. Error bars represent robust standard deviation from triplicate staining conditions from a single cell culture. (c) GFP fluorescence in unstained untransfected FreeStyle 293-F cells and FreeStyle 293-F cells transiently transfected with Na_V_1.7_IRES-GFP. (d) Cy5 fluorescence in untransfected and Na_V_1.7_IRES-GFP-transfected FreeStyle 293-F cells that were unstained or stained with 10, 100, or 1000 nM R4C8-Cy5. Histograms show live, single cells, with MFI indicated for each population.

**Figure S14.** Additional electrophysiological analysis for R4C8 at Na_V_1.7. (a) Current-voltage (I–V) relationships from HEK293-Na_V_1.7/β1 cells in control (black) and after application of 10 μM R4C8 (blue) (n = 4). (b) Summary of V_50_ of activation from HEK293-Na_V_1.7/β1 cells showing no statistically significant differences between control and R4C8 (P = 0.147, paired two-tailed Student’s t-test). Individual points represent biological replicates. (c) Summary of V_50_ of steady-state fast inactivation from HEK293-Na_V_1.7/β1 showing a statistically significant difference between control and R4C8 (P = 0.021, paired two-tailed Student’s t-test). Individual points represent biological replicate. (d) Representative sodium currents from *Xenopus* oocytes expressing Na_V_1.7 in control conditions (black) and after application of 10 µM R4C8 (green). Currents elicited by a voltage pulse to –10 mV are shown in orange. (e) Current-voltage (I–V) relationship from Na_V_1.7 expressed in oocytes in control (black) and with 10 µM R4C8 (green) (n = 8). (f) Summary of V_50_ of activation from Na_V_1.7 in oocytes showing no statistically significant differences between control and R4C8 (P = 0.378, two-tailed paired Student’s t-test). Individual data points represent separate oocytes. (g) Summary of V_50_ of steady-state fast inactivation from Na_V_1.7 in oocytes showing no statistically significant differences between control and R4C8 (P = 0.567, two-tailed paired Student’s t-test). Individual data points represent separate oocytes. Data in panels a and e are presented as average ± SEM; panels b, c, f, and g show individual data points with lines indicating average ± SEM.

**Figure S15.** Negative controls for R4C8-HA staining of primary human chondrocytes. Representative confocal images of chondrocytes stained with the anti-HA primary and fluorescent secondary antibodies in the absence of R4C8-HA (a), or incubated with R4C8-HA without either detection antibody (b). For each condition, the merged fluorescence and DAPI image is shown on the left and the fluorescence channel alone on the right. Scale bars: 20 μm.

**Table S1.** Full-length sequence of nanobodies (with His_6_ tag) generated against p-loop 1.

| ID | Sequence |
| --- | --- |
| R3D3 | QVQLQESGGGLVQPGGSLRLSCLVSGKLSNYSAIGWIRQAPGKEDEGVSCISGGGEESYHTDPVKGRFTISKDNDKNTVYLQMDNLKPEDTALYRCAADGDVTARGMCVIPYADYWGQGTQVTVSSHHHHHH |
| R3D8 | QVQLQESGGGLVQDGGSLRLSCVASGFIFDDYAIGWFRQAPGKEREFVAAISWNGLSTRYADSVKGRFTISRDNAKIAVYLQMDNLTPDDTARYYCTMRAWSENTGNFASRGQGTQVTVSSHHHHHH |
| R3E6 | QVQLQESGGGLVQPGESLRLSCVASTSDFSLYNMGWYRQAPGKEREFVAAISWTGGSTYYADSVKGRFTISRDTAKNTLYLQMNSLKSEDTAVYYCKFAGAPWGQGTQVTVSSHHHHHH |
| R3F8 | QVQLQESGGGLVQPGGSLRLSCTASGFTFGKAGMSWVRQSPGKGLEWVATIFPSSGNTYYIDSVKGRFTISKDNSKNTLYLQMNNLQPEDTAVYHCATALSVYGCVPPRYWGQGTQVTVSSHHHHHH |
| R3H9 | QVQLQESGGGLVQPGGSLRLSCSVSGKLSNYSAIGWIRQVPGKNDEGVSCISGGGEETYHTDLVQGRFTISKDIDKNIVYLQMDDLKPEDTALYRCAADGDVTALGMCVIPYADYYGQGTQVTVSSHHHHHH |
| R4A5 | QVQLQESGGGLVQPGGSLRLSCVASGISADFYHLGWFRQAPGKEREFIAAISWTGGSTYYADSVKGRFTISRDNAENTVDLQMNSLKSEDTAVYYCNGIKGGRPLGPWGQGTQVTVSSHHHHHH |
| R4A7 | QVQLQESGGGLVEPGGSLRLSCAASGRTFSSYVMGWFRQAPGKERELVAAISKSGGSTYYADSVKGRSTISRDNAKNTVYLQMNSLKPEDTAVYYCNTGRHWGKGTQVTVSSHHHHHH |
| R4B9 | QVQLQESGGGLAQAGGSLRLSCTVSGGTFSSYPMGWFRQAPGKERESVAAISYSGNTTWYADSVKGRFSISRDNAKNMLYLQMNSLKPEDTALYYCATGQYYYTDHRQGILGQGTQVTVSSHHHHHH |
| R4C8 | QVQLQESGGGLVPPGGSLTLSCAVSEFSSDLKAIGWFRQAPEKEREGVAAIATHRYQDGVTYYRESVKGRFTISRNSANHTVSLQMNDLKPDDTAVYFCNTVPIQDWGRGTQVTVSSHHHHHH |
| R4C9 | QVQLQESGGGLVQAGGSLRLSCAASGRTFSTYTMAWFRQVPGNERELVTAISWSGKSTWYADSVKGRFTISRNNAKNTAYLQMNNLKPEDTAVYSCAAGSRAERLTMGNGDYWGQGTQVTVSSHHHHHH |
| R4D3 | QVQLQESGGGSVQAGGSLRLSCAASGGTFSTYHMAWFRQAPGKEREFVAAIAGSGGLTSYGDSVKGRFTISRDNAKNTVSLQLNSLKPEDTALYYCARSPQGWGGAMDYWGKGTQVTVSSHHHHHH |
| R4E9 | QVQLQESGGGLVQSGGSLRLSCTASGFNLQYWPVGWFRQAPGKEREWVSAISSGGGMTYYADSVKGRFTISRDNAKNTLFLQMNSLKAEDTAVYYCATGHRTNRMDYWGKGTQVTVSSHHHHHH |
| R4F12 | QVQLQESGGGLVQAGNSLRLACAASGLTFSSYAMGWFRQAPGKEREFVAAISGSGVVTKYADSVKGRFTISRDNRKNTVFLQMNSLKPEDTAVYYCTARGVMSNYWGQGTQVTVSSHHHHHH |
| R4G10 | QVQLQESGGGLVQAGGSLRLSCAASGRTFSSYVMGWFRQAPGKERELVAAISKSGGSTYYADSVKGRSTISRDNAKSTVYLQMNSLKPEDTAVYYCNTGASWGQGTQVTVSSHHHHHH |

**Table S2.** Initial nanobody candidate triage.

| **Nanobody** | **Library source** | **HSQC behaviour** | **AF2 ipTM** | **AF2 PAE** | **Outcome** |
| --- | --- | --- | --- | --- | --- |
| R3D3 | Immunization | Weak/no binding | 0.327 | 15.7 | Not Advanced |
| R3D8 | Immunization | Strong binding | 0.825 | 7.4 | Advanced |
| R3E6 | Immunization | Excluded | ND | ND | Not Advanced |
| R3F8 | Immunization | Aggregation | 0.625 | 10.8 | Not Advanced |
| R3H9 | Immunization | Weak/no binding | 0.533 | 12.3 | Not Advanced |
| R4A5 | Proprietary VHH | Excluded | ND | ND | Not Advanced |
| R4A7 | Proprietary VHH | Strong binding | 0.708 | 9.42 | Advanced |
| R4B9 | Proprietary VHH | Aggregation | 0.449 | 14.3 | Not Advanced |
| R4C8 | Proprietary VHH | Strong binding | 0.759 | 9.3 | Advanced |
| R4C9 | Proprietary VHH | Strong binding | 0.696 | 13.3 | Not Advanced |
| R4D3 | Proprietary VHH | Weak/no binding | 0.599 | 11.8 | Not Advanced |
| R4E9 | Proprietary VHH | Aggregation | 0.36 | 15.0 | Not Advanced |
| R4F12 | Proprietary VHH | Strong binding | 0.844 | 6.5 | Advanced |
| R4G10 | Proprietary VHH | Strong binding | 0.476 | 12.5 | Not Advanced |

ND is Not Determined

**Table S3.** Summary of crystallographic structure determination statistics.

| Data collection statistics | R3D8 – P-loop1 |
| --- | --- |
| PDB ID | 9Y7F |
| Space group | P 1 21 1 |
| Resolution (Å) | 43.64 – 1.70  (1.73 – 1.70) |
| a, b, c (Å)  α, β, γ (°) | 38.83, 43.64, 42.33  90.00, 107.84, 90.00 |
| Total reflections | 29,454 (2,771) |
| Unique reflections | 14,873 (1,392) |
| Completeness (%) | 99.3 (89.6) |
| Rmerge+ | 0.091 (0.874) |
| Rpim* | 0.058 (0.571) |
| CC1/2 | 0.995 (0.580) |
| <I/σ(I)> | 9.9 (1.50) |
| Multiplicity | 6.5 (6.0) |
| Molecule/asym | 1 |
| Refinement statistics |  |
| Rwork/Rfree (%) ¶# | 20.58/24.06 |
| No. protein atoms | 1,205 |
| Waters | 82 |
| Wilson B (Å2) | 23.27 |
| Average B (Å2)^ | 28.22 |
| Protein | 27.97 |
| Water | 31.79 |
| rmsd bonds (Å) | 0.009 |
| rmsd angles (°) | 1.056 |
| Ramachandran plot: |  |
| Favored/outliers (%) | 95.86/1.38 |

Values in parentheses refer to the highest resolution shell. +Rmerge = Σ|I – <I> | ⁄ Σ<I>, where I is the intensity of each individual reflection. *Rpim indicates all I+ & I-. ¶Rwork = Σh|Fo - Fc|/ Σl| Fo|, where Fo and Fc are the observed and calculated structure-factor amplitudes for each reflection h. #Rfree was calculated with 10% of the diffraction data selected randomly and excluded from refinement. ^Calculated using Baverage.

**Table S4.** Characterisation of lead nanobody candidates.

| **Nanobody** | **ITC *K*_D_ for**  **p-loop 1 (nM)** | **Predicted orientation** | **zz-exchange support** | **Crystal structure** | **Cellular recognition** |
| --- | --- | --- | --- | --- | --- |
| R3D8 | 64.4 ± 7.6 | Channel-facing/occluded | Channel-facing | R3D8:p-loop 1 | ND |
| R4A7 | 256.3 ± 14.1 | Channel-facing/occluded | Channel-facing | ND | ND |
| R4C8 | 86.5 ± 8.0 | Extracellular-accessible | Accessible surface | ND | Yes |
| R4F12 | 67.3 ± 3.2 | Channel-facing/occluded | Channel-facing | ND | ND |

ND: Not Determined

**Table S5.** Image-analysis summary for the R4C8 Na_V_ subtype-selectivity staining panel.

| **Cell Line** | **Area** | **Cy5 IntDen**  **(whole image)** | **Background (mean)** | | | **Corrected Cy5 IntDen**  **(whole image)** | **Cell numbers** | **Signal per cell** |
| --- | --- | --- | --- | --- | --- | --- | --- | --- |
|  |  |  | **1** | **2** | **3** |  |  |  |
| HEK293 | 18211.82 | 9485.30 | 0.15 | 0.38 | 0.06 | 5915.78 | 20 | 295.79 |
| hNa_V_1.1 | 18211.82 | 65.17 | 0.00 | 0.00 | 0.00 | 65.17 | 68 | 0.96 |
| hNa_V_1.2 | 18211.82 | 86.01 | 0.00 | 0.00 | 0.00 | 86.01 | 82 | 1.05 |
| hNa_V_1.3 | 18211.82 | 0.07 | 0.00 | 0.00 | 0.00 | 0.07 | 36 | 0.00 |
| hNa_V_1.4 | 18211.82 | 124.36 | 0.00 | 0.00 | 0.00 | 124.36 | 41 | 3.03 |
| hNa_V_1.5 | 18211.82 | 0.56 | 0.00 | 0.00 | 0.00 | 0.56 | 46 | 0.01 |
| hNa_V_1.6 | 18211.82 | 5673.20 | 0.00 | 0.08 | 0.00 | 5193.62 | 53 | 97.99 |
| hNa_V_1.7_1 | 18211.82 | 49302.15 | 0.10 | 0.15 | 0.17 | 46788.92 | 39 | 1199.72 |
| hNa_V_1.7_2 | 249214.43 | 316257.80 | 0.00 | 0.00 | 0.00 | 316091.66 | 180 | 1756.06 |
| hNa_V_1.8 | 18211.82 | 182.44 | 0.00 | 0.00 | 0.00 | 177.54 | 10 | 17.75 |
| MsNa_V_1.7_1 | 18211.82 | 5377.11 | 0.06 | 0.02 | 0.11 | 4211.55 | 7 | 601.65 |
| MsNa_V_1.7_2 | 18211.82 | 9790.63 | 0.03 | 0.41 | 0.02 | 6992.08 | 18 | 388.45 |

**Supplementary References**

[1]	E. Pardon, T. Laeremans, S. Triest, S. G. Rasmussen, A. Wohlkönig, A. Ruf, S. Muyldermans, W. G. Hol, B. K. Kobilka, J. Steyaert, *Nat Protoc* **2014**, *9*, 674-693.

[2]	aM. Mobli, *J Magn Reson* **2015**, *256*, 60-69; bM. Mobli, M. W. Maciejewski, M. R. Gryk, J. C. Hoch, *Nat Methods* **2007**, *4*, 467-468; cM. Mobli, T. M. Miljenovic, *J Magn Reson* **2019**, *300*, 103-113.

[3]	Y. Shen, A. Bax, *Methods Mol Biol* **2015**, *1260*, 17-32.

[4]	W. F. Vranken, W. Boucher, T. J. Stevens, R. H. Fogh, A. Pajon, M. Llinas, E. L. Ulrich, J. L. Markley, J. Ionides, E. D. Laue, *Proteins* **2005**, *59*, 687-696.

[5]	P. R. Evans, G. N. Murshudov, *Acta Crystallogr D* **2013**, *69*, 1204-1214.

[6]	A. J. Mccoy, R. W. Grosse-Kunstleve, P. D. Adams, M. D. Winn, L. C. Storoni, R. J. Read, *J Appl Crystallogr* **2007**, *40*, 658-674.

[7]	P. D. Adams, P. V. Afonine, G. Bunkoczi, V. B. Chen, I. W. Davis, N. Echols, J. J. Headd, L. W. Hung, G. J. Kapral, R. W. Grosse-Kunstleve, A. J. McCoy, N. W. Moriarty, R. Oeffner, R. J. Read, D. C. Richardson, J. S. Richardson, T. C. Terwilliger, P. H. Zwart, *Acta Crystallogr D Biol Crystallogr* **2010**, *66*, 213-221.

[8]	V. B. Chen, W. B. Arendall, 3rd, J. J. Headd, D. A. Keedy, R. M. Immormino, G. J. Kapral, L. W. Murray, J. S. Richardson, D. C. Richardson, *Acta Crystallogr D Biol Crystallogr* **2010**, *66*, 12-21.

[9]	A. Mueller, Z. Dekan, Q. Kaas, A. J. Agwa, H. Starobova, P. F. Alewood, C. I. Schroeder, M. Mobli, J. R. Deuis, I. Vetter, *ACS Pharmacol Transl Sci* **2020**, *3*, 535-546.

[10]	aS. Wakale, Y. Chen, A. R. Sun, C. Liyanage, J. Gunter, J. Batra, R. Crawford, H. Sang, I. Prasadam, *Cell Prolif* **2025**, *58*, e13776; bX. Wu, C. Liyanage, M. Plan, T. Stark, T. McCubbin, R. A. Barrero, J. Batra, R. Crawford, Y. Xiao, I. Prasadam, *Osteoarthritis Cartilage* **2023**, *31*, 613-626.

[11]	F. Madeira, N. Madhusoodanan, J. Lee, A. Eusebi, A. Niewielska, A. R. N. Tivey, R. Lopez, S. Butcher, *Nucleic Acids Res* **2024**, *52*, W521-w525.

[12]	C. D. Livingstone, G. J. Barton, *Comput Appl Biosci* **1993**, *9*, 745-756.

[13]	J. Jumper, R. Evans, A. Pritzel, T. Green, M. Figurnov, O. Ronneberger, K. Tunyasuvunakool, R. Bates, A. Žídek, A. Potapenko, A. Bridgland, C. Meyer, S. A. A. Kohl, A. J. Ballard, A. Cowie, B. Romera-Paredes, S. Nikolov, R. Jain, J. Adler, T. Back, S. Petersen, D. Reiman, E. Clancy, M. Zielinski, M. Steinegger, M. Pacholska, T. Berghammer, S. Bodenstein, D. Silver, O. Vinyals, A. W. Senior, K. Kavukcuoglu, P. Kohli, D. Hassabis, *Nature* **2021**, *596*, 583-589.
